# “The hypothesis becomes forced”: Cultural narratives about deductive hypothesis testing influence scientists’ self-perception and actions

**DOI:** 10.1371/journal.pone.0346386

**Published:** 2026-08-21

**Authors:** Hailey Fazio, R. H. Khediri, Nicole M. James

**Affiliations:** Chemistry Department, Reed College, Portland, Oregon, United States of America; Yantai Institute of Technology, CHINA

## Abstract

There is a rich variety of approaches for generating scientific knowledge. However, deductive hypothesis testing is often considered a defining characteristic of the scientific method. It has long been shown that individuals’ perceptions of how science works often do not align with the actual nature of science, which can adversely impact scientific progress and scientific literacy. Here we present an emergent study exploring how postdoctoral scholars whose work involves materials and chemistry describe the role of hypothesis testing in their work. Through a reflexive thematic analysis of 16 interviews, we highlight how participants broadly report valuing hypothesis-based approaches and view them to be a defining characteristic of “good” science. However, simultaneously, a portion of participants find that deductive hypothesis testing is not always the most practical or useful approach in their work. This dissonance between their value of deductive hypothesis testing and their own scientific practices has implications for how participants view themselves and their work. We argue that the cultural master narrative of science emphasizes the use of deductive hypothesis-based reasoning, and that confronting incompatibilities with this narrative requires participants to formulate alternative narratives to rationalize their work in light of the cultural master narrative. This process influences participants’ self-perception, their scientific practices, and/or their scientific communication. These findings illustrate that explicit consideration of the nature of science continues to be important in higher education, and motivate further study in this area. These findings also reinforce existing calls for educators and scientists to explicitly normalize the existence of multiple legitimate approaches for generating scientific knowledge.

## Introduction

Science is a process for developing knowledge about the world. Because this can be done in many ways, there is no singular definition of the essential characteristics that make something “science” or “not science” [1]. However, in contemporary science, hypotheses are often considered a necessary and axiomatic pillar of science [2–5]. Yet, there is also no singular definition of what constitutes a hypothesis [2]. Rather, there exist multiple formulations or types of hypotheses used to generate knowledge [6,7]. Hypothesis-based scientific reasoning can follow deductive, inductive, and abductive approaches [4,8,9]. The deductivist approach [10], also referred to as the hypothetico-deductive method (HDM) [7], follows a top-down and often iterative process where a falsifiable hypothesis is postulated to generate claims that can be empirically tested through systematic data collection and analysis, which may refute the hypothesis [11].

The HDM is often viewed as the defining characteristic or “common denominator” that distinguishes science from non-science [10–12]. In this way, the HDM has been treated as the “gold standard” in science [5,13]. For example: research is often perceived as more rigorous when it is presented as employing the HDM; consequently, HDM-driven studies are more likely to be supplied with resources such as funding [14,15].

It is important to note that this has long been contested as impossible [16], as well as historically and currently untrue [4,7,17,18]. Other approaches or “styles” to scientific knowledge generation exist and are often used [19], such as in applied or discovery-based science [20]. For example, in areas where empirical data is abundant and/or generalized theories are less prevalent, inductive data-driven approaches may be more common [5,8,14,18]. Yet, the association between the HDM and scientific rigor is pervasive, and can incentivize practices such as retroactively presenting work as though the project had followed the HDM (i.e., been driven *a priori* by a hypothesis), when in fact the study involved inductive hypotheses (i.e., hypotheses developed *post hoc,* from analysis of the results) [21].

The presentation of a *post hoc* (inductive) hypothesis as if it were an *a priori* (deductive) hypothesis is referred to as “HARKing”: hypothesizing after results are known [21]. Kerr argues that presenting an inductive *post hoc* hypothesis as if it were a deductive *a priori* hypothesis can negatively affect the quality of findings and impair scientific discovery and theory building. Others have similarly condemned HARKing [22–24], argued it is a form of sharpshooter fallacy [25], and advocated for cultural and/or systemic changes in how science is conducted to reduce incentives for HARKing [26–28]. Concurrently, some have emphasized the difference between “secretive” HARKing and “transparent” HARKing, often questioning the integrity of secretive HARKing [29] and detailing the value of transparent HARKing [30–32]. However, others have also argued that secretive HARKing may not necessarily have negative effects [33], or the existence of negative impacts is unclear and requires further study [34].

The association between deductive hypothesis testing methods and rigor is common throughout the physical, biological, and social sciences. However, much of the discussion about data-driven work and HARKing has focused on biological and social science contexts. Here we extend this discussion by reporting emergent findings about how researchers whose work involves materials and chemistry describe their use of hypothesis testing.

Materials science is a highly interdisciplinary field focused on understanding, characterizing, and designing material properties [35,36]. Materials science is rapidly growing [37,38] and provides tools for addressing urgent global challenges, such as sustainable energy solutions [39]. Materials science is recognized not as a single discipline, but a “multidisciplinary matrix of those disciplines which are related through the structure/property/process/function/performance linkage of materials” [40,p.169]. As a result, most researchers conducting materials-related work are formally trained in other natural science disciplines, such as chemistry or physics [40–42].

Here we report emergent findings encountered during the process of an ongoing mixed-methods project that aims to characterize the skills and concepts used by postdoctoral scholars (postdocs) whose work involves materials and chemistry. A component of this project included semi-structured interviews, where participants described the role of hypothesis testing in their work. In response, participants described rich and complex characteristics of their broader scientific practices and approach. This prompted us to re-analyze these data using reflexive thematic analysis to address the research questions:

1. How do participants view hypothesis testing in scientific work?
2. How do these views influence how participants approach or perceive their work?

Here we focused specifically on postdocs to maximize participants’ research experience while still targeting the experiences of individuals who are embedded in the details and day-to-day realities of experimentation, data collection, and data analysis in their projects. The focus on materials and chemistry was driven by the ongoing project’s aims. However, we argue this is a particularly interesting domain to examine perspectives on nature of science because the multi- and inter-disciplinary nature of the materials science/chemistry interface suggests that individuals in our sample are likely to have experience with multiple disciplinary cultures, norms, and traditions, which may offer unique insights and perspectives about the nature of science.

## Theoretical foundations

In this study, we employ a critical realist and contextualist epistemology to structure the study design. This informed our selection of frameworks and methods that enable us to capture participants’ perceptions of reality, and to allow space for how this perception may be influenced by broader structures and systems of science and society. As this was an emergent project, within this critical realist lens we inductively selected theoretical frameworks to guide our analysis based on our initial consideration of participant responses. Because participants tended to describe their hypothesis use in the context of their broader scientific approach to generating knowledge, we draw on the Nature of Science scholarship to structure our analysis and interpretations. We draw on both the consensus view of the nature of science to afford us a fine-grained perspective of the process of science, as well as the revised family resemblance approach to afford us a structure for considering the role of broader structures and systems in science and society. When asked about their use of hypothesis testing, participants often discussed personal, social, or systemic expectations in and about science in general. Thus, we also draw on the scholarship on master cultural narratives to guide our thinking about participants’ impressions and associations of science, and of their own scientific practices.

### Nature of science

The Nature of Science (NoS) describes core features of the epistemological foundations of science (i.e., how knowledge is constructed), and the associated values and beliefs [43–45]. There is no singular definition of NoS, but there is a “consensus view” on core features [45–47]. In brief: this includes how science is a creative endeavor with various ways to generate knowledge about natural phenomena, largely through analyzing and drawing inferences from observations and empirical evidence, which generates durable yet tentative knowledge that is tested and iteratively revised. The consensus view also emphasizes that science is performed by people, and thus science is influenced by researchers’ imagination and reasoning, and is embedded in social and cultural traditions. Consequently, while scientific efforts may strive to minimize subjectivity, they can never be entirely objective.

The consensus view is not without critique [48,49], and alternative formulations exist. For example, some formulations aim to contextualize the NoS more explicitly within sociocultural contexts [50,51]. The “family resemblance approach” describes overlapping features that are broadly common throughout the sciences, explicitly recognizing that individual disciplines may use a subset or alternative construction of these features [52,53]. The “reconceptualized” family resemblance approach specifically aims to facilitate use in science education [54,55].

It is important to recognize that implicitly-developed views about science often conflict with established tenets of NoS, such as the common belief that science is objective [46]. Partial or biased conceptions about NoS are often reflected in and perpetuated by science curricula [56–58] and scientific systems [49]. All models, but especially the family resemblance approach, acknowledge there may be variation in specific scientific methods and approaches. Thus, partial formulations of the NoS may accurately reflect the NoS in a specific disciplinary context. However, extrapolating these discipline-specific formulations beyond the scope of the discipline to describe science at large would conflict with these broader NoS models.

**Fig 1:**
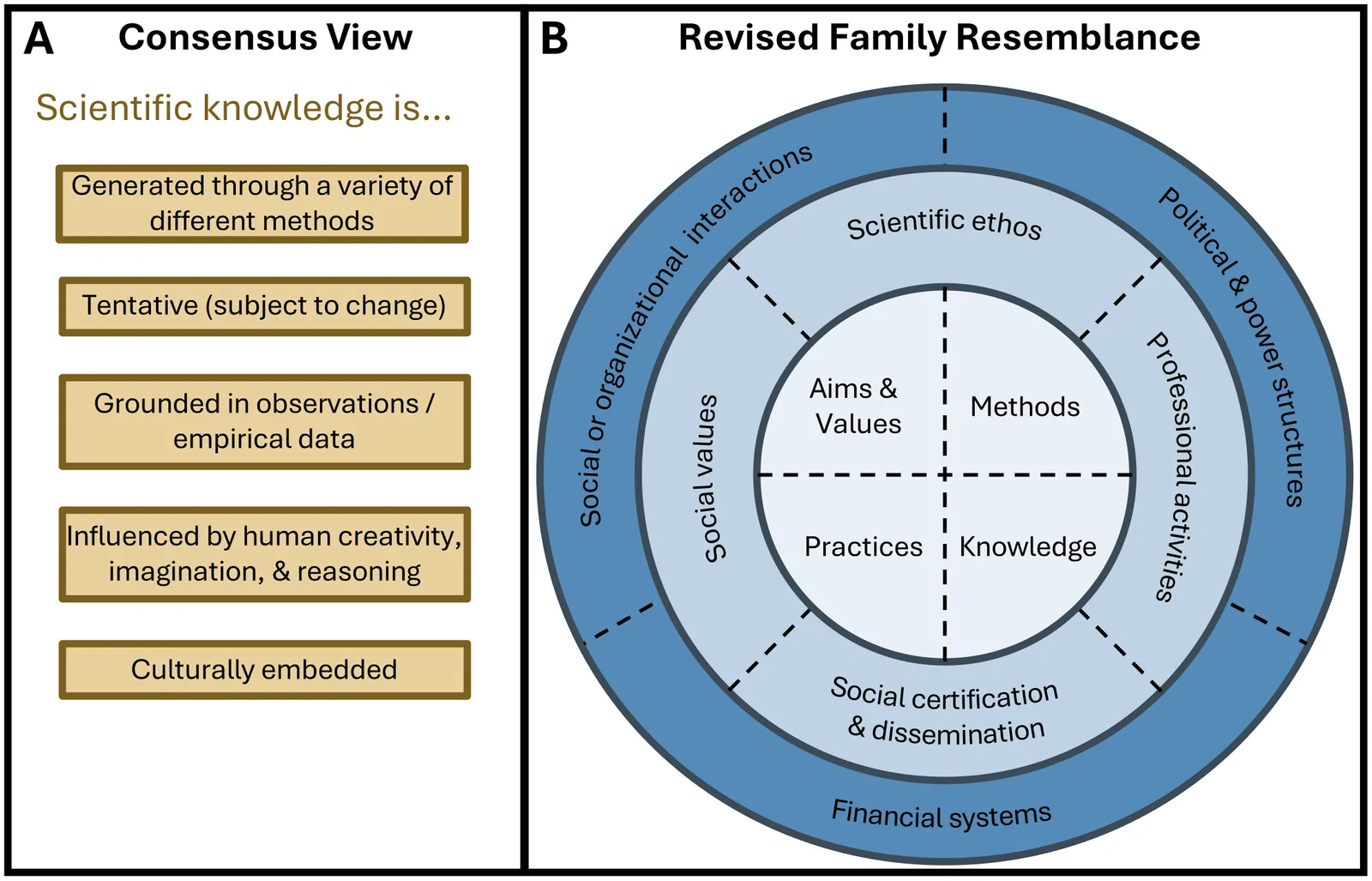
Summary of key features of the consensus view NoS and revised family resemblance approach to the NoS. We primarily draw on the consensus view (A) to structure our thinking about the NoS practices employed by study participants. In order to ensure we acknowledge how the NoS may vary across disciplines and under the influence of broader societal institutions and structures, we supplement this theoretical understanding with the revised family resemblance approach to the NoS (B, adapted from [50]). For the purposes of this study, we view these models to be complementary and mutually compatible.

For the purposes of this study, to operationalize our conception of science we draw primarily on the NoS consensus view. However, aligned with our critical realist epistemology, to acknowledge the broader systems and structures that influence individuals’ actions and experiences, we also draw on the revised family resemblance approach. Fig 1 summarizes the features of these models that we draw on to structure our thinking. In particular: we recognize that participants likely hold varied conceptions about science, and differences in conceptions may reflect disciplinary differences in practices. However, it is possible that individuals’ conceptions do not align with reality but nonetheless influence their practices and thinking. Furthermore, these implicit and explicit concepts and thought processes occur under the influence of broader institutional and societal structures.

### Cultural master narratives of science

Because the NoS may not accurately reflect individuals’ perceptions of science, we also draw on the cultural master narratives scholarship [59] in our analysis. Cultural master narratives (CMNs) are pervasive, culturally shared stories that inform one’s thoughts, beliefs, behaviors, and values [59,60]. Master narratives and cultural master narratives have been widely used to describe how individuals construct and negotiate their identities [59–64]. McLean & Syed’s CMN framework was developed to explain identity construction and details how individuals’ personal identities are influenced by dominant master narratives [59]. To briefly summarize: McLean & Syed outline five key principles of cultural master narratives: *ubiquity, invisibility, utility, rigidity*, and *compulsory nature*. These principles describe how cultural master narratives exist ubiquitously at societal and cultural levels, often operating as an implicit (invisible) frame. Cultural master narratives structure how individuals understand the position of groups in society—and features are internalized and used to define oneself, often based on alignment or opposition with membership in particular social and cultural groups. This primarily occurs unconsciously and implicitly, with individuals not realizing how heavily their self-perceptions are influenced by master narratives. While cultural narratives are socially constructed and thus dynamic, cultural master narratives are relatively rigid and enduring attributes of the sociocultural milieu. Critically, cultural master narratives also carry a “compulsory nature”: aligning with the cultural master narrative relays benefits, such as being viewed as “good” or “right.” When individuals’ experiences diverge from or are inconsistent with the master narrative, this can greatly impact their self-perceptions and identities [61,64]. A complex negotiation process can result in the construction of alternative narratives that allow one to rationalize and accommodate both their personal narrative and the master narrative.

During our initial analysis, we realized CMN may be a relevant and useful lens for structuring our interpretations, particularly because of features we noticed (see: Results) that suggested participants may hold implicit conceptions of “good” science that are heavily connected to the HDM, and that participants’ own (mis-)alignment with these models bore implications for their self-perception and identity as scientists. Through iteratively re-examining the existing CMN and NoS literature, we recognized attributes of cultural master narrative principles (*utility, ubiquity, invisibility, rigidity, and compulsory nature*) about the nature of science, which we refer to as cultural master narratives of science (CMNoS).

To be clear: this is a conceptual framework [65] developed for the purposes of this project’s analysis. To operationalize the CMNoS for this purpose, we attended to areas in the literature that have shown how students’ and practicing scientists’ conceptions of science conflict with NoS, especially in areas that are highly conserved across multiple educational levels. In particular, this includes the conception that science objectively reveals absolute truths [66] and operates through definitively proving or disproving hypotheses [20]. The development of these conceptions is reinforced by subtle factors, such as the nature of teachers’ language [67] and the lack of explicit instruction on the nature of science [46]. This is consistent with how cultural master narratives are often unstated and learned implicitly [59].

In some cases, students’ views of NoS may develop with age [68]. However, conceptions of science as axiomatically objective and hypothesis-driven commonly persist [69] and can become more pronounced over time [70]. Throughout many studies, the conception of deductive hypothesis testing as a sole or defining characteristic of science is well-conserved [44,71–73]. This can be seen, for example, through the widely-held conception that science is a structured step-by-step (linear) empirical process, often following the HDM. This conception has been observed in teachers [74,75], high school students [76], undergraduate students [77,78], graduate students [78], and doctoral scientists [79–81]. Thus, while NoS conceptions vary greatly—including among practicing scientists [81]—the relative consistency with which deductive hypothesis-based approaches are referred to or provided as illustrative examples of scientific work underscores its durability in the cultural narrative of what science is and how science works.

In this way, here we consider a central feature of the CMNoS to be the generalization of systematic deductive hypothesis testing, i.e., the hypothetico-deductive model (HDM), as a universal defining characteristic of science. This aligns with all five principles of cultural master narratives: it is widely present in existing NoS literature (*ubiquity*), often implicit or indirectly discussed (*invisibility*), and yet is a tangible and enduring component of reported science conceptions across decades, disciplines, and educational levels (*rigidity*). Furthermore, it facilitates describing science and the behavior of scientists (*utility*), and has implications for identifying “good” science and allocating material benefits such as funding and recognition (*compulsory nature*).

**Fig 2:**
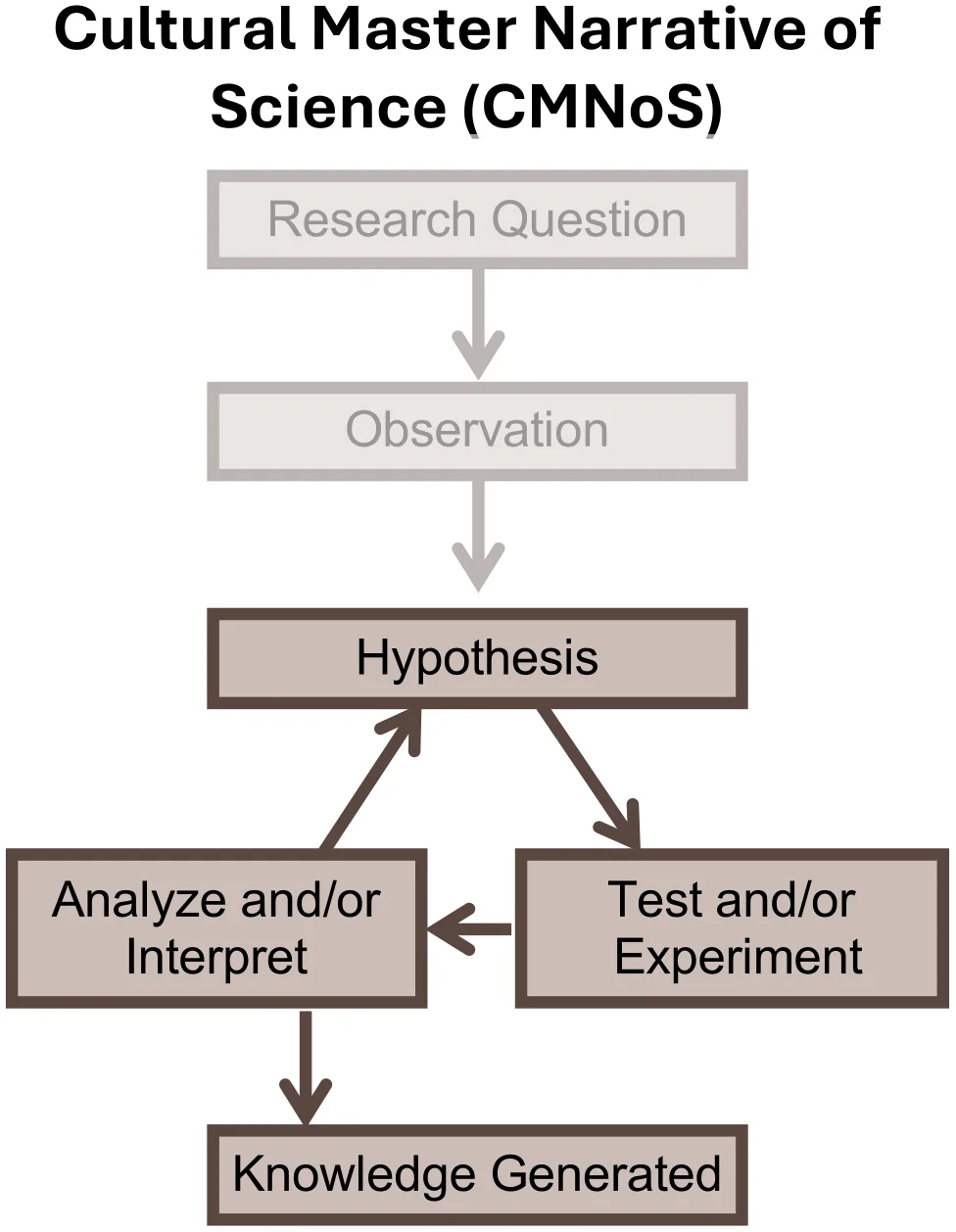
Summary of the key components of the cultural master narrative of science (CMNoS) conceptual framework developed to inform this study. Throughout the literature, the hypothetico-deductive approach—illustrated by the iterative process of hypothesis formulation, experimentation/hypothesis testing and analysis/interpretation of collected data—often presents as a universal defining feature of science, abstracted from other components of the NoS such as creativity and cultural embeddedness (see Fig 1). The less prevalent presence of science as a structured linear process involving research question formulation and observation is represented here by illustrating these as linearly-connected, semi-transparent features.

In sum, for the purposes of this study we draw on the NoS and CMN literature to formulate the cultural master narrative of science (CMNoS) conceptual framework. The CMNoS reflects the conception that science can be universally described as an objective pursuit that involves iterative and systematic deductive testing of hypotheses, i.e., the hypothetico-deductive model. The preceding stages of research question formulation and observation are sometimes present or implied, often in the context of science being a structured linear process (see Fig 2). Importantly, the CMNoS does **not** accurately reflect reality as described by the consensus view and revised family resemblance models of NoS. Instead, the CMNoS overgeneralizes one narrow and partial view of the nature of science, extrapolating it to be a defining characteristic of all science. We argue that recognizing the existence of this inaccurate but pervasive narrative is relevant for analyzing and interpreting participants’ narratives about science. Furthermore, the existing scholarship around cultural master narratives enables us to draw inferences about how these narratives can influence individuals’ scientific practices and identities.

## Methods

This study was approved as exempt by the Reed College Institutional Review Board (#2022-S33). All participants were adults who provided audio-visually recorded verbal consent to participate, and provided additional separate verbal consent for their data to be re-analyzed as part of future studies.

### Recruitment

To be eligible, participants must be a current US-based postdoctoral scholar who self-described their work to involve materials and chemistry at the time of participation. We compiled a list of postdoctoral scholars listed on institutional websites as affiliated with materials science research centers, and whose institutional email addresses were publicly available. We used a random number generator to randomly select invited participants and staggered invitations over time to facilitate preliminary analysis of the data alongside data collection. Participant recruitment began on February 6, 2023 and ended on July 8, 2024.

These data are a subset of the data collected and analyzed for a separate study. Toward that study’s aims, we noticed signs of saturation (e.g., reinforcing existing interpretations and not yielding new information outside that scope) during preliminary data analysis of the 13^th^ interview, which did not change upon collection and preliminary analysis of the next three interviews. Thus, data collection concluded following the 16^th^ interview (*n = 16*)*.*

### Participants

All participants (*n* = 16) were postdoctoral scholars who described their work to involve materials and chemistry. Because context, backgrounds, and identities influence experiences and perceptions, we include a summary of participants’ demographic and related information (Table 1). All names are pseudonyms; participants could select their own pseudonym or request to have one assigned.

**Table 1:** Participant demographic information. Participants’ self-reported prior scientific training includes Chemistry or Chemical Engineering (CHEM), Materials Science or Materials Engineering (MAT), Biology (BIO), Physics (PHYS), and/or Engineering (ENG).

Pseudonym	Gender	Race/Ethnicity	Institution Type	Experience	Prior Training
Loui	Male	Asian or Asian American	Public R1	4–6 years	CHEM
Maya	Female	Asian or Asian American, Other	Public R1	<1 year	CHEM
Iris	Female	Hispanic, Latinx, or Spanish	Public R1	>6 years	ENG, MAT
Melody	Female	Asian or Asian American	Research Institute or Government Agency	1–3 years	MAT
Jon	Male	Asian or Asian American	Private R1	4–6 years	CHEM, MAT
Mark	Male	White or Caucasian	Research Institute or Government Agency	4–6 years	CHEM
Zayn	Male	Asian or Asian American	Private R1	4–6 years	CHEM, PHYS
Slovenia	Female	White or Caucasian	Private R1	>6 years	CHEM
Maxmillian	Male	White or Caucasian	Public R1	>6 years	CHEM, PHYS
Reuben	Male	Asian or Asian American	Private R1	1–3 years	BIO, PHYS, ENG
Thor	Male	White or Caucasian	Private R1	4–6 years	PHYS
Tom	Male	Asian or Asian American	Private R1	4–6 years	CHEM, MAT
James	Male	Asian or Asian American	Public R1	>6 years	MAT, PHYS
Lily	Female	White or Caucasian	Public R1	>6 years	MAT
Taylor	Non-binary	White or Caucasian	Public R1	>6 years	PHYS
Henri	Male	Asian or Asian American	Private R1	>6 years	MAT

In the interview, participants were asked to describe their research area or sub-discipline. Collectively, participants described their area using one or more of the following terms: 2D materials, active materials, additive manufacturing, biopolymers, catalysis, computational, condensed matter, hybrid materials, nanoparticles, nanomaterials, polymers, self-assembly, sensor development, semiconductors, soft matter, solid-state inorganic materials, ultrafast spectroscopy.

Note: We do not include disaggregated research area/subdiscipline information in Table 1 because participants tended to avoid specific “labels” for their area or sub-discipline, instead providing extended descriptions of the types of work they did that—if reproduced—may render them identifiable to others in their community. In follow-up questions to clarify the nature of their sub-discipline, participants either rejected the idea that there existed a way to “label” their research area, or provided several labels—with participants often stating that they used different terms to describe their area depending on contextual factors such as the audience’s background (i.e., whom they are talking to). Multiple participants expressed that this stems from the inherently interdisciplinary nature of their work. As such, we feel it would be misleading to infer sub-disciplines for each participant. However, we believe the aggregate summary of these field descriptors is a more useful and meaningful characterization of the study sample as a whole.

### Data collection

This emergent study analyzes qualitative data originally collected as part of a larger, separate study. Participants engaged in 30–45 minute semi-structured Zoom interviews about the skills and concepts used in their work. Co-author N.M.J. served as lead interviewer in all interviews, supported by a secondary undergraduate interviewer who served as an active listener and occasionally asked clarifying or follow-up questions. Participants’ time was compensated with $50 via their choice of Amazon gift card, Venmo, or Zelle. Interviews were audio-video recorded, transcribed by a transcription service, and checked for accuracy by comparison to recordings.

Participants were provided a summary of the interview questions in advance, which included questions about the laboratory and data analysis techniques they use, as well as the concepts, uncertainty considerations, and strategies they use to develop and support research conclusions. The full interview protocol included in Supporting Information. Before data collection, a preliminary draft of this protocol was tested and iteratively refined through four pilot interviews. In this study, we focused specifically on participants’ responses to the question: **“Could you talk a bit about the role hypothesis testing plays in your work?”** For data transparency, de-identified excerpts of participant responses to this question can be found in the Supporting Information. We consulted the full interview transcript and recording as necessary to understand participants’ responses to this question. All researchers working with this data were concurrently working on the original study this work emerged from, such that the work presented in this publication required no additional data access to be granted.

### Data analysis

Both primary and secondary interviewers recorded independent analytical memos immediately after conducting the interview and then met to discuss overall impressions. In these discussions, we noted complex themes in participants’ discussion of their hypothesis use. This was beyond the scope of the original study, which motivated us to re-analyze these data as a separate study (this publication). Similar to a grounded-theory approach, our framework selection (see: Theoretical Foundations) and research question development were informed by these preliminary stages of memoing and consensus discussion.

Informed by these frameworks, to address our research questions, we conducted a reflexive thematic analysis [82,83]. The often-implicit nature of cultural master narratives and the nature of participant descriptions motivated us to employ an interpretive reflexive thematic analysis [84], which did not involve codebook development or the use of software other than typical word processing software (e.g., Google Docs, Microsoft Word). First, co-author H.F. reviewed all interview recordings and transcripts. Then, N.M.J. and H.F. each independently read the hypothesis question portion of all interview transcripts, independently recording analytical memos. H.F. and N.M.J. then discussed their interpretations, collaboratively generating a set of preliminary themes. H.F. then drafted initial 1-page descriptions of each theme, with supportive evidence from interview recordings and transcripts. These initial descriptions were revised iteratively through discussions and collaborative editing, with H.F. and N.M.J. often referring back to interview recordings and other sections of the transcripts to substantiate interpretations. After drafting the final description of the themes, with supporting quotes from interview transcripts, N.M.J. reviewed the full transcripts from all interviews for disconfirming evidence and found none.

### Quality considerations

To ensure the rigor and quality of this analysis, we attended to the trustworthiness criteria of credibility, transferability, dependability, and confirmability [85,86]. Dependability was addressed by maintaining a detailed documentation trail of data collection methods (e.g., interview protocol) and reasoning (e.g., analytical memos) throughout the project. Credibility was supported by researchers’ independent analysis and collaborative consensus discussions, as well as through triangulating themes across multiple participants. To make this triangulation more visible to the reader, throughout the manuscript, we routinely include illustrative quotes from multiple participants. Credibility was further supported by re-examining all collected data for disconfirming evidence.

Informed by the literature in this area [87,88], we elected not to solicit participants to engage in member checking of transcripts or analysis. Based on our theoretical foundations, we anticipate many participants’ conceptions of the nature of science are implicit, and may change over time following the type of explicit consideration that participation in this study prompted for some participants. Furthermore, this work aims to generate a collective understanding, beyond the level of any one participant. For these reasons, to establish credibility we instead rely on multi-level triangulation and examining the data for disconfirming cases.

Confirmability was attended to through reflexivity and examining the consistency of findings with existing peer-reviewed literature (e.g., nature of science, cultural master narratives). As part of this reflexivity, we intentionally considered our research paradigm and have included an explicit description of how we drew on the literature to guide our analysis. We also recognize that our prior experiences in science shape how we collect, interpret, and notice features of data. In particular, co-author N.M.J.’s doctoral research focused on discovery-based materials chemistry research. This insight was an asset in data collection and analysis, but also presents the possibility of bias. To minimize this bias, N.M.J. was intentional to refer frequently back to the data sources for evidence supporting interpretations, and collaboratively discussed all interpretations to consensus with H.F., who holds a bachelor’s degree in psychology. During this project, H.F. was a post-bachelor research assistant working under the mentorship of N.M.J. To minimize the influence of powder dynamics within the research team, the team began this project with an explicit discussion of the importance of asking questions and sharing all thoughts, especially alternative or contradicting interpretations. Additionally, to minimize the likelihood of H.F. unconsciously revising their interpretations after hearing N.M.J.’s interpretations, in all consensus discussions and analysis conversations, H.F. was invited to share their interpretations and reasoning first.

There is evidence that scientific practices will vary greatly between individuals and research fields. Consequently, to support transferability, we report detailed demographic information that includes elements of participants’ academic background, such as institution type, scientific training, and length of experience. We also summarize and/or include participants’ descriptions of contextual factors of their research areas, which influence their practices and perceptions.

### Limits and scope

Here we outline aspects of this study that influence the limits and scope of these findings. Importantly, this is an emergent study: features we noticed in data collection prompted our retrospective re-analysis of collected data. Had we designed data collection with these research questions in mind *a priori*, we likely would have asked different interview questions, which would have influenced the data analyzed here. Consequently, this study does not capture contextual mechanisms that may have influenced the development of participants’ views of science, their scientific practices, and their science identity. Thus, our findings are limited to describing features present in participants’ responses to primarily one question about their use of hypothesis testing, wherein participants often described their scientific practices and views about science. Thus, we acknowledge that factors not captured here likely also contribute to participants’ views. For example, there is reason to expect that scientific approaches will vary as a function of one’s disciplinary and sub-disciplinary experience and training. However, as noted in our description of the study participants, there is significant fluidity in how participants identify or describe their research area and training—which they often attribute to the interdisciplinary nature of their work at the materials and chemistry interface. For these reasons, we are not able to resolve disciplinary and sub-disciplinary patterns in these data.

This study is an inductive investigation into participants’ descriptions of how they do their work. Importantly, what participants describe doing *in practice* may differ from what they believe one “should” do *ideally*. In this study, we cannot and do not wish to comment on or evaluate the quality of participants’ work. Rather, we operate under the assumption that, as practicing postdocs, these participants are successful scientists and we report themes in their self-reported “real life” practices.

As reported in existing literature, scientific practices vary greatly based on the nature of the work and the culture of individual disciplines or subfields. Participants in this study self-identified their work to involve materials and chemistry, but their professional backgrounds (e.g., professional training, years’ experience, etc.) are highly varied. This provides a rich and broad landscape for qualitative analysis, but we note that these themes will not manifest in the same ways or extents in all fields. However, within their context, we view these findings to provide new insights that extend existing scholarship about the nature of science, with implications for science education and practice in higher education.

## Results

Through reflexive thematic analysis we identified five themes, summarized in Table 2. In the following sections, we describe the multifaceted nature of each theme individually. In the Discussion section, we expand on the overlaps and relationships between themes, and their implications.

**Table 2:** Summary of characteristics of each theme.

Theme	Characteristics
Hypotheses as defining characteristics of (good) science	Pervasive perceptions of hypotheses and hypothesis testing as important in their approach to their work, and as a defining characteristic of how science should be done.
Hypotheses are not always the most relevant tool	Perceptions of how some scientific work does not lend itself to being structured, approached, or described in terms of hypothesis testing.
Exploratory work is important, but is it enough?	Perceptions of exploratory or science as valuable and essential, alongside implicit or explicit reference to external forces that privilege hypothesis-based approaches
Are you really a scientist if your work isn’t hypothesis-driven?	Indicators of nervousness, self-consciousness, and/or discomfort when participants struggle to describe their work in terms of hypotheses and hypothesis testing.
Personal feelings and inclinations influence participants’ work	Descriptions of how affective factors and implicit or unconscious knowledge (e.g., curiosity, intuition) drive participants’ work, in contrast to hypothesis-based approaches.

### Hypotheses as defining characteristics of (good) science

This theme represents how participants often emphasized the importance of hypothesis testing in science, generally framing “good” science as requiring a hypothesis. Some participants described hypothesis testing as the core framework that guides how they approach their projects. For example:

“[...] so we need to make hypothesis to move forward our project. Yeah. So that’s really important, but it should be based on our basic knowledge.” (Loui)

“So I am personally a huge believer in hypothesis testing. So I try to design pretty much all of my experiments around the hypothesis and using a hypothesis testing method.” (Iris)

“So the hypothesis testing at the beginning of the project is also very important in order for you to design the studies, to design the experiment, and that will construct the later on of the project.” (Reuben)

Additionally, participants often described hypothesis testing as a core component of what science is or how science should be done. For example (emphasis added):

“I think hypothesis testing is something **very important, not only in my work but in science in general**.” (Reuben)

“I think **hypothesis testing is exactly [...] how the research is kind of driven** and how I like to do research too [...] You start by kind of reading what’s there already and how people are envisioning things to be and you find where the gaps are, and then you make a hypothesis” (Jon)

“So we prepare to write something down in a mathematical way. So if we have some hypothesis, that means we have some equation, right? And we want to explain some experimental issues by using the equation, right? [...] I think **this kind of approach is important in our field**.” (James)

In this way, participants described hypotheses not only as a valued approach they use in their work, but also frame it as a central, defining characteristic of what science is and how science should be done. While not clearly stated, these descriptions often imply a deductive hypothesis formulation of hypothesis testing.

### Hypotheses are not always the most relevant tool

This theme describes how the specific context and aims of participants’ work may make other approaches more useful or practical than the hypothetico-deductive method (HDM). For some participants, this is because their work is largely application-driven:

“It’s definitely more of a kind of engineering viewpoint on the research where you’re trying to design a specific thing. [...] where it’s then hypothesis driven, I guess, is that you look at an outcome and you think like, ‘Oh, if I change these aspects of my structure, then that will push me more towards this kind of desired target.’ But a lot of it is more design oriented” (*Thor*)

Alternatively, this could be because the participant’s approach is primarily data-driven. For example:

“I wouldn’t personally choose hypothesis testing as the way to describe it because, essentially, you're never getting to really the full classical loop of hypothesis testing evaluation to new hypothesis. It’s really, I'm looking at some data, I get a sense of there’s a pattern, and so you can sort of fill in the details. [...] I'm certainly not wedded to any particular explanation.” (*Taylor*)

Other participants describe the HDM as not helpful for their work because formulating a deductive hypothesis relies on having a theoretical or empirical knowledge base that is not practical in their area. For example:

“[...] so much of the field is so messy. While things are broadly reproducible, everyone’s nanocrystal is slightly different from lab to lab and from person to person, even in the same lab. And so a lot of it is like ground-truthing what you do know before you can start speculating.” (*Zayn*)

“for example, development of new materials for electronics, you simply would not have the theory to predict everything that’s going to work or not. And in the absence of that, the hypothesis becomes forced [...] this is coming from someone who is in a very experimental field. So the answer to this will be very different if you talk to someone who’s doing - I don’t know - core physics or something, right? Then the theories are very well developed. [...] there are countless examples of things that should work that don’t work for practical reasons. And the practical reasons are never incorporated in a theory because there are too many of them. It doesn’t make sense to think about [...] I think if you try to do it in a very systematic way, the tradeoff would be you would not be able to try as many things.” (Henri)

Along similar lines, Lily says

“I have lots of hypotheses, and I'm often very, very wrong. But usually, it’s in an interesting way, so that works. I do a lot more, I think, of framing research questions because I'm wrong so often [...] So I'll be like, ‘Well, I want to know fundamentally how this type of structure impacts this kind of response.’ And then I spend a lot of time thinking about: How do I want to study that? How do I design the experiments that will give me confidence in the data that will actually answer the question that I'm setting out to understand or confirm my hypothesis, if I have one?” (*Lily*)

Lily’s description in particular shows how hypothesis use is not “all or nothing,” and researchers may fluidly switch between using deductive and inductive hypothesis-based approaches alongside other approaches, based on their perceptions of what is most effective for their project or goal at that moment.

Taken together, these examples illustrate how the HDM is one class of approach to scientific practice, which can be useful in many contexts. However, it is not the only approach, and researchers may not perceive it to be useful for all scientific work.

### Exploratory work is important, but is it enough?

This theme reflects how participants describe tensions between exploratory scientific work and the use of the HDM. Commonly, participants describe their work as fundamentally requiring exploratory approaches, but often frame this in a way that implies they or others do not view exploratory work to be sufficiently “scientific” unless it is associated with a hypothesis. For example, Iris says:

“[...] there is also a fair bit of exploration in my work where it’s just, let’s try things and see what happens. [...] when something interesting emerges, then we're going to take the hypothesis method of really testing and understanding why is this happening [...] So the starting point is a lot of kind of just exploration-gotten intuition. But at least for me personally, I really try to employ that hypothesis testing method for all of my subsequent like, oh, not just what’s the random cool thing I found, but what’s the actual science that I'm going to put and understand behind this?” (*Iris*)

Here, Iris described an initial exploratory stage of her project that eventually led to a hypothesis-testing stage. This full arc is reminiscent of inductive hypothesis-based approaches. However, Iris’ description could be taken to imply that–while essential–the exploratory stage is primarily a means for reaching the hypothesis stage, which is viewed as more legitimate “actual science.” This framing suggests that while exploratory work is essential, it may be viewed as less scientific than hypothesis-based work.

In contrast, when asked about the role hypothesis testing plays in his work, Zayn says:

“I think people like to pitch academic science as a Socratic kind of investigation, where it ends up being Edisonian a lot of times. It’s kind of like, ‘Hey, let me throw this in the pot and see what happens,’ rather than formulating a formal hypothesis and then testing. So I would say that I think an idealized picture is one where you hypothesis test. I think a more realistic picture is where you have a known system, and then you go to set out to do five modifications, of which one may work or yield something interesting. And then […] [in] the paper, obviously, you write it like you had that hypothesis. [...] that’s very much the paradigm under which I operate.” (Zayn)

Unlike Iris, Zayn appears to perceive that his “actual science” is inherently exploratory, involving trial and error without developing into a hypothesis-based investigation stage. However, Zayn’s reference to “obviously” writing the paper as though a hypothesis was involved illustrates how he recognizes the norm or expectation that published work involves a deductive hypothesis.

In a similar vein, when discussing the role of hypothesis testing in his work, Henri says:

"I think at some point, you have to make a compromise between how much hypothesis-based research you want to do and how many new things you want to do. Because sometimes [...] you discover penicillin by accident." (*Henri*)

In response to a follow-up question about this, Henri says:

“You can tune the composition of your material to get the property that you want based on [...] intuition [...] and then you go to a collaborator and you ask them, ‘Does this make sense?’ And then it does, right? [...] and then the way you write it in a paper, is that we did that thing first, and then we made this, right? But what had happened was you had the intuition first, and then you asked someone to check it Then that was correct. And then you use that for your experiments. But the first point was the intuition. It was not the hypothesis. It was not the theoretical hypothesis.” (*Henri*)

Here, Henri also appears to view his work as fundamentally exploratory and discovery-based work; he does not seem to personally believe that a hypothesis is necessary or must drive the project for it to be “good science.” However, like Zayn, Henri also acknowledges there is a norm or expectation to write the resulting publication as though the project employed a deductive hypothesis, even when it did not.

In summary, participants described exploratory-driven work as valuable and important, while also recognizing a cultural expectation (i.e., cultural master narrative) that scientific work use hypothesis-based approaches that are largely deductive in nature.

### Are you really a scientist if your work isn’t hypothesis-driven?

This theme reflects the affective impacts participants communicated verbally or through observable body language when discussing how their work involved hypothesis testing. Notably, this study examines responses to one question asked within the context of a larger semi-structured interview. Participants who spoke freely and readily in response to earlier questions would often hesitate substantially upon reaching this question, or make facial expressions that suggested uncertainty and/or self-consciousness. Some participants also made verbal comments explicitly indicating uncertainty. For example:

“Yeah, this is the one question I wasn’t sure how to answer from the list you sent me [laughter].” (*Slovenia*)

To the research team, this laughter seemed to indicate nervousness. Slovenia goes on to say:

“Well, of course, for example, in the current project that I have, I hypothesize that I'm able to combine the [two components] into a hybrid material, and then I can do this in different ways. For instance, I can grow the organic material in situ on the inorganic support, or I can pre-synthesize both materials and then try to combine them post-synthetically. So these are the kind of, I think, hypotheses that I test. [...] Yeah, so there definitely is a lot of hypothesis testing, right?” (*Slovenia*)

However, she later adds:

“[hypotheses inform] smaller sections of the project, yeah. I feel like maybe the bigger hypothesis, overarching the whole project, is more applicable to biological studies or biochemistry, maybe. You're hypothesizing this protein in this pathway will do this and that, and then if you remove it, this and that will happen. Maybe. I don’t know. Maybe it’s a wrong feeling. Yeah.” (*Slovenia*)

From these comments, we can see that Slovenia reported using hypotheses when they are useful for subcomponents of her projects, but did not report using a deductive hypothesis to structure her work. She displayed nervousness in answering the question, initially using hypothesis-based language before concluding that a formal hypothetico-deductive approach didn’t lend itself to her work. This nervousness and initial attempt to “fit” her project into an HDM framework suggests an initial implicit impression that her work “should” align with the HDM.

In response to this same question, Taylor says:

“honestly, I think I'm still not quite late enough in my career for [hypotheses] to play a particularly large role in my own work. […] I think I have been more exploratory, I wouldn’t personally choose hypothesis testing as the way to describe it because, essentially, you’re never going to really get the full classical loop of hypothesis testing evaluation to new hypothesis. It’s really, I'm looking at some data, I get a sense of there’s a pattern, and so you can sort of fill in the details. But I don’t have a particular-- I probably don’t have an explanation yet, or I'm certainly not wedded to any particular explanation” (*Taylor*)

Taylor does not visibly appear nervous or self-conscious when responding to this question. However, attributing the lack of hypothesis testing in their work to not being “late enough” in their career implies that the use of the HDM is indicative of one’s scientific “maturity.” Notably, at the time of this interview, Taylor was a postdoc with a doctorate and 6+ years of experience in their field.

At the same time, Taylor’s description reveals that their work is grounded in a discovery-based, exploratory approach, involving iterative engagement with data, pattern recognition, and refinement of ideas. While they resist labeling this process as hypothesis testing, their account reflects a rigorous, data-driven methodology. Importantly, Taylor’s attribution of hypothesis testing to “mature” scientific work implies that exploratory approaches are “immature” or preliminary. This framing not only reflects internalized disciplinary hierarchies but also illustrates how perceptions of scientific legitimacy may be closely tied to method.

In summary, some participants display indicators of nervousness, self-consciousness, and/or discomfort when asked how hypothesis testing manifests in their work. This influences how they describe their work (e.g., in an effort to align it with the HDM) or themselves (e.g., their development or status as scientists).

### Personal feelings and inclinations influence participants’ work

This theme reflects how participants described affective factors such as enthusiasm, intuition, and curiosity to influence their work. For example:

“So most of it’s driven by my great enthusiasm for microstructures and kind of really understanding what’s going on at the atomic level. So that, and I’m just really easily excited by any context, any concept, whatever in materials.” (*Iris*)

“I think just my curiosity [drives my projects]. I think it’s very different for maybe a first-year

grad student versus a postdoc who’s been in it for awhile. Kind of you leave your PhD with a couple ideas that you're like: ‘Oh, I wish I could have worked on that,’ or like, ‘thought about this’ or, ‘I'm just curious about what will happen.’” (*Zayn*)

“And we don’t really – we are not an application-oriented research, but I also kind of love to think from a fundamental, a curiosity-driven rather than application driven.” (*Tom*)

Across participants, curiosity emerged as a key motivator for their work. This was expressed both by individuals engaged in exploratory research and by those who reported using the HDM. For example, while Iris previously described herself as a strong proponent of systematic hypothesis testing, she also emphasizes how affective factors like curiosity and enthusiasm motivate her work. Similarly, in follow-up questions asking participants how they plan or make decisions about the direction of their projects without a hypothesis, participants often described affective factors:

“For me, it’s a very creative process and a certain amount of it does come down to just let’s give it a try” (*Iris*)

“I balance my desire to persevere with a recognition that not everything works the way I expect it to sometimes, and I won’t always know why. So if things don’t go as planned, maybe I’ll try it another two or three times. And if that doesn’t work after that, there are so many other questions to answer scientifically that it’s pretty easy to move on to a different thing.” (*Mark*)

“Intuition more than anything, I think. So from what I’ve seen – so again, this is coming from someone in a very experimental field […] I think the best researchers that I work with, the common trait that they have is they have this intuition for what should and should not work, rather than a strong theoretical background about what should and should not work because there are countless examples of things that should work that don’t work for practical reasons.” (*Henri*)

In this way, participants describe affective factors such as intuition and creativity as valuable tools in navigating the uncertainty and complexities in their work.

In summary, this theme highlights how personal feelings and affective factors such as curiosity, enthusiasm, intuition, and creativity play an influential role in participants’ scientific decision-making.

## Discussion

These themes are highly interconnected, and together collectively address our stated research questions. At the center, the theme **Hypotheses as defining characteristics of (good) science** reflects the cultural master narrative that hypotheses are necessary attributes of “good” science. Aligned with the *ubiquity* principle of cultural master narratives, this is a highly prevalent sentiment among participants*,* seen in cases such as Iris and Reuben who expressed a strong personal value of the hypothetico-deductive model (HDM). However, this is directly complicated by the nature of their work, as highlighted in the theme **Hypotheses are not always the most relevant tool**. This theme represents how the disciplinary context, project aim, or available theory base may limit the usefulness of the HDM. For example, Thor and Maxmillian describe their work to be more goal-oriented and iterative, making a formal *a priori* hypothesis impractical. Other participants, such as Zayn and Henri, who engage in more discovery-based, exploratory work also highlight the limited usefulness of *a priori* hypothesis-based approaches for their context and purposes. These accounts suggest that while participants recognize deductive hypothesis testing as a component of the CMNoS, the nature of their work requires different practices. These participants often propose alternative narratives to justify or accommodate the nature of their work in light of the CMNoS. For example, Iris goes through a negotiation process, initially trying to fit her work into the HDM structure of the CMNoS, before ultimately justifying her deviation from the CMNoS. Henri rationalized his deviation from the CMNoS based on differences in his field’s available theory base and his personal interest in focusing more on discovery-based work (i.e., doing “new things”).

When individuals’ identities, lives, and/or experiences differ from cultural master narratives, they construct alternative narratives; in doing so, they often question and/or feel a need to justify themselves [59]. This can be seen in the theme **Exploratory work is important, but is it enough?** Some participants describe exploratory work as essential, but still recognize the legitimacy associated with HDM-driven inquiry in the CMNoS. This reflects the *rigidity* and *compulsory nature* of cultural master narratives. For example, Iris’s description of how initial exploratory stages build up into hypothesis-driven stages implies that the hypothesis is an important component of what makes her feel that the work is “actual science.” In contrast, Zayn is critical of the necessity of deductive hypotheses in his work. However, he recognizes that a deductive hypothesis is expected by others (per the CMNoS), which can be seen in how he reports “obviously” presenting his work as though there were a deductive, *a priori* hypothesis.

This negotiation of exploratory work and hypothesis-driven work influences participants’ self-perception as scientists, reflected in the theme **Are you really a scientist if your work isn’t hypothesis-driven?** The internalization of the CMNoS, and participants’ deviation from the CMNoS, produces feelings of hesitation, discomfort, or even inadequacy. For example: Slovenia’s nervous laughter and negotiation process reflect an effort to align her work with the HDM, despite acknowledging that her approach does not follow that structure consistently. Similarly, Taylor attributes their non-use of the HDM to being early-career, suggesting an association between the HDM and scientific rigor or maturity. These moments highlight how the CMNoS implicitly shapes self-perceptions of scientific legitimacy, in alignment with the cultural master narrative *invisibility* principle.

Participants’ perceptions of themselves influences their work, as reflected in the theme **Personal feelings and inclinations influence participants’ work**. This theme reflects how emotions and personal dispositions shape participants’ work. For example, Iris and Zayn describe how their enthusiasm and curiosity informs their research directions, and Henri describes a good sense of intuition as essential for his work. This is well aligned with the NoS, which describes science as a creative and social process. However, this is not conserved in the CMNoS, which associates “good” science with being strictly structured and objective.

Overall, these themes highlight a dissonance between which scientific epistemologies and approaches are valued and legitimized by the CMNoS, and which are perceived by some participants to be most relevant for their work. As seen throughout these themes, this dissonance influences how participants view themselves and their work. For some participants, this prompts a complex negotiation process with participants justifying or rationalizing their scientific practices relative to the CMNoS. Sometimes, this negotiation process leads participants to discount portions of their scientific process and/or question their own status or “maturity” as scientists.

### Implications

This work further supports calls raised by prior studies to examine how science is presented societally and in science education. Even though NoS recognizes deductive hypothesis testing as one tool among many in the scientific toolkit, the cultural master narrative of science (CMNoS) implicitly but tangibly frames “good” science as an objective pursuit axiomatically defined by use of deductive hypothesis testing. Here we find this deeply and often unconsciously internalized, including by practicing post-doctoral scientists whose own work does not conform to this model. Thus, we argue it is important for STEM instruction in higher education to explicitly recognize and normalize science as a holistic and multifaceted approach inherently influenced by affective factors such as creativity, curiosity, and intuition. The CMNoS’s positioning of “good” science as objective suggests that these affective factors are “bad” or unscientific. Rather, consistent with scholarship about the NoS and the experiences described by participants in this study, we suggest that creativity, curiosity, and intuition can be assets in scientific work when accompanied by critical reflection and examination of how one’s scientific arguments are supported by their analysis of data. This raises questions about how we talk about science and teach scientific practices at the undergraduate, graduate, and post-doctoral level.

This work highlights how the CMNoS influences scientists’ experience and self-perceptions, which has implications for inclusivity and scientific progress. This raises many questions beyond the scope of this study, but we emphasize the importance of continuing investigations in this area. For example: this finding raises concerns that students and future scientists may be making career decisions based on partial or misleading information about what it would mean for them to “do science.” If this is true, then students who may have been enthusiastic and successful in some forms of science, such as exploratory or discovery-based work, may be dissuaded from pursuing science of any form because of its (disproportionate) association with the HDM. Alternatively, students who are drawn to the HDM may unintentionally pursue areas of science where it is not a widely practical tool (e.g., exploratory or application-driven work), and the misalignment between their interests and the nature of that work may prompt them to leave science altogether.

In this way, these findings emphasize that existing established knowledge about the nature of science is also highly relevant for higher education. In this way, this work reinforces existing calls to explicitly embrace the multiplicity of scientific approaches, and highlights how this may better support future scientists and promote a more honest and inclusive scientific culture.

## Conclusions

In this article, we report findings from a reflexive thematic analysis of interviews with 16 postdoctoral researchers whose work involves materials and chemistry. We identified themes that describe how participants recognized and responded to the existence of cultural master narratives of science (CMNoS) that position objectivity and deductive *a priori* hypothesis-based approaches as a defining characteristics of rigorous science. While often implicit or unconscious, participants displayed awareness—and often internalization—of the CMNoS. Characteristics of the CMNoS are useful in certain contexts and situations, and some participants’ work aligned well with the CMNoS. However, for other participants, the CMNoS does not well accommodate the needs of their personal and disciplinary contexts, particularly for participants whose work is exploratory, discovery-based, or application-driven. These features are well-captured by established scholarship about the nature of science (NoS), which highlights science as a creative pursuit that can employ a variety of abductive, inductive, and deductive methods for establishing knowledge about the world. However, recognizing and negotiating differences between the CMNoS and participants’ actual scientific experience and practice can adversely influence participants’ practices and self-perceptions.

These findings emphasize the importance of attending to long-standing calls to improve recognition of the multifaceted and holistic nature of science, and echo warnings of harm that can result when this is not done. While such calls are often focused on K-12 education and how science is communicated to the general public, this study highlights that these same features remain important areas of attention in higher education, as they continue to impact practicing post-doctoral scientists.
