# Supplementary material for: “The hypothesis becomes forced”: Cultural narratives about deductive hypothesis testing influence scientists’ self-perception and actions
Source: PLoS One. 2026 Aug 21;21(8):e0346386. doi: 10.1371/journal.pone.0346386 (PMC13497266; doi:10.1371/journal.pone.0346386)
Supplement: Supplementary file 1 [file pone.0346386.s003.pdf]

Response to Review Comments for Manuscript PONE-D-25-36217

Dear Editor and Reviewers,

Thank you for your thoughtful review of our submitted manuscript. Below, please find our responses (in blue) to your comments (in black).

Editor Comments

E-1. We note that there is identifying data in the Table 1. […] Please remove or anonymize all personal information (Name), ensure that the data shared are in accordance with participant consent […]

The data in Table 1 was fully anonymized; as described in text, the names listed were pseudonyms; participants were asked to select their own pseudonym and had the option to request to have one assigned. We have clarified this directly in Table 1 replacing the "Name" column header with the title "Pseudonym".

E-2. Standardize terminology, clarify analytic stance, and expand description of coding and theme development.

We believe our changes made throughout the manuscript in response to the other comments have greatly standardized the terminology used in the manuscript, and further clarified the analytical stance.

As described in our original submission, we employed reflexive thematic analysis, rigorously following the methods established by Braun and Clark (Braun & Clarke 2006; Braun & Clark 2019; Braun & Clarke 2021a; Braun & Clarke 2021b). We emphasize that there are many approaches to thematic analysis (TA), each of which may employ researcher reflexivity. However, "TA with reflexivity" is distinct from "reflexive TA" as developed by Braun and Clarke. Reflexive TA, following Braun and Clarke, does not demand—and in some circumstances cautions against—employing a codebook development and coding process.

We have endeavored to clarify this further by explicitly stating that we did not employ a coding process, alongside our description of how themes were generated (p. 8, clean copy of revised manuscript)

E-3. Provide a clear Sampling & Recruitment subsection, including rationale for sample size.

We have added this section (p. 6, clean copy of revised manuscript).

E-4. Specify interview language, transcription workflow, and interviewer roles. Add a reflexive note on power dynamics.

We have further emphasized our inclusion of the full interview protocol in the supporting information, and added a summary of the nature of interview prompts (p. 8, clean copy of revised manuscript), added further information to the interviewer roles and explanation of the transcription process (p. 8, clean copy of revised manuscript), and described how the team handled power dynamics in data analysis (p. 9, clean copy of revised manuscript).

E-5. Clarify stance on member checking, state if software was used, and include a theme table (main text or supplementary).

We have explained why we elected not to employ member checking (p. 9, clean copy of revised manuscript), explicitly stated our non-use of CAQDAS software (p. 8, clean copy of revised manuscript), and added a theme table to the main text (p. 10, clean copy of revised manuscript)

E-6. A COREQ checklist should be completed and uploaded to ensure full methodological transparency.

We have completed a COREQ checklist and included it as part of the Supporting Information.

E-7. The reference list appropriately includes foundational sources but relies heavily on older literature (>5 years). Recent publications (2020–2025) are included but could be strengthened further by incorporating the latest scholarship on:

- Qualitative reporting standards in higher education research. (for Qualitative reporting follow these studies and cite them; https://doi.org/10.1186/s12909-025-07185-9 https://doi.org/10.1155/2024/1271802)
- Researcher identity and methodological culture in STEM postdocs.
- Current debates around hypothesis testing, openness, and HARKing.

In the submitted manuscript, we do follow and cite two recent publications on qualitative reporting standards (Johnson et al 2020; Stahl & King 2020), as well as two publications more specifically about the thematic analysis approach—and associated data reporting standards—used in this work (Braun & Clarke, 2019; Braun & Clarke, 2021). Following discussion with the journal office (case number 09407575), we have decided not to cite the two recommended references.

We have added additional references to further support our discussion of hypothesis

testing, transparency, and HARKing (p 2, clean copy of revised manuscript): (Hu et al., 2023), (Cockburn et al. 2020),.

E-8. Ensure consistent abbreviations (e.g., HDM vs HDMs)

Our prior manuscript used HDMs when the text was used in sentences that resulted in a plural (i.e., "methods" instead of "method), and had defined this in our first use of the plural term and abbreviation. However, to align all abbreviations, we have reworded sentences to refer to "method" in the singular, thus removing all instances of "HDMs".

E-9. Consider adding a visual model illustrating (if possible)

We presume this comment referred to a model illustrating the relationship between themes. Based on the nature of the data analyzed in this emergent study, we hesitate to do this. Rather, we prefer to position the themes themselves as the primary result of the paper, and explicate the relationships, overlap, and interactions between themes in the discussion section. While we find these to be suggested by and highly consistent with the data, we hesitate to highlight these in a figure—which would be the only figure in the manuscript. We fear this may lead readers to misinterpret the relative support these data give to differing elements of the manuscript. As described in the Methods > Limits and Scope section, this is an emergent study and re-analysis of data that was originally collected to address other research questions. While we believe all our claims are supported by the data, we believe the strongest claims are the themes themselves, and prefer to position the relationships of themes more as discussion of this result. We believe these relationships are still well-communicated in text, and fear the nuance of these points may be lost to readers who may not closely read this discussion of the figure.

That said, we recognize the value in having a visual summary of the main results, and feel the theme table (Table 2, p. 10, clean copy of revised manuscript), which was added in response to other comments, addresses this need.

Reviewer 1 Comments

R1-1. Line 71; It would be helpful if the authors could provide a clearer rationale for focusing on postdoctoral researchers in materials and chemistry. In particular, connecting this choice to specific aspects of the nature of science, or to the unique modes of thinking, paradigms, and experimental approaches in materials and chemistry, would enhance the contribution of the study.

We have added a paragraph addressing this to the introduction (p. 3, clean copy of revised manuscript)

R1-2. Lines 91 and 562; The research questions are clearly stated; however, in the Discussion section, I recommend adding concise answers to each research question. It may also strengthen the clarity of the discussion if the subheadings correspond explicitly to these research questions.

We appreciate the value in this and appreciate the reviewer raising this point. In our careful consideration of this suggestion, we have grappled with how the answers to our research questions ("1. How do participants view hypothesis testing in scientific work?; 2. How do these views influence how participants approach or perceive their work?") are interrelated and nuanced. We are concerned that incorporating more concise and stand-alone sections dedicated to these research questions individually will obscure these interrelationships and nuance, and may run the risk of portraying participants as monolithic.

Rather, we believe the collective discussion of the relationships between themes best addresses our research questions. To make this connection to our research questions clearer, we have removed the section subheading ("Relationship between themes"), and revised the first sentence of the discussion section, which now reads: "These themes are highly interconnected, and together collectively address our stated research questions" (p. 17, clean copy of revised manuscript).

R1-3. Line 224; The description of the interview content could be elaborated further. Please specify what additional questions were asked beyond those already mentioned, so that the reader can better understand the scope of the interviews.

We agree with the reviewer that transparency about the full interview protocol is highly important. In our original submission, the full interview protocol can be found in the supplementary information, which was stated in the Methods > Data Collection section. To further increase transparency around this, we have added a brief description of the nature of the other questions in the interview protocol. This section now reads: "*Participants were provided a summary of the interview questions in advance, which included questions about the laboratory and data analysis techniques they use in their work, as well as the concepts, uncertainty considerations, and strategies for developing and supporting their research conclusions. The full interview protocol is included in the Supporting Information*." (p. 8, clean copy of revised manuscript).

R1-4. Line 520; Table 1 presents information on Experience and Prior Training. Were there no noteworthy patterns or features related to these variables? If none were observed, a brief statement clarifying this would improve the completeness of the discussion.

We agree that this is a natural question to ask! Given the nature of this study design and our sample, we hesitate to draw patterns from these variables, as we suspect there is likely significant variability in the nature of participants' Experience and Prior Training even for participants with the same number of years' of experience disciplinary training—and, as detailed in response to Reviewer's Comment 2 (R2-2)—it is not as simple as one might expect to define what sub-discipline each participant is working in, which is likely also

relevant context here. Given this "blurriness" and variation, we think it is not feasible to detect patterns with confidence in this area. We had added a description of this to the "Limits and Scope" section (p. 10, clean copy of revised manuscript)

Reviewer 2 Comments

R2-1 The introduction currently frames HDM within "deductivist approaches" and treats it as the paradigmatic ("gold standard") model of scientific inquiry. Please clarify the inferential architecture of HDM and justify its placement under a purely deductive banner. […] HDM, as widely construed in contemporary philosophy of science and science education discourse, involves a composite workflow […] The manuscript would benefit from an explicit account of these phases and of why you classify HDM as 'deductive,' given this mixed-mode character. If you retain a deductivist classification, please present principled criteria for that choice and acknowledge alternative taxonomies used in the literature. […]Requested revision: Insert a dedicated subsection (in Introduction or Theoretical Foundations) that (i) delineates HDM's mixed inferential structure, (ii) justifies your deductivist labeling, and (iii) offers a brief historically grounded rationale for HDM's dominance and linkage to CMNoS.

We appreciate the author raising this point, as it highlights that our intention here can be easily misinterpreted. We are not aiming to argue that science is *rightly* defined by deductivist approaches or the HDM. Rather, our intention is to frame science as inherently multifaceted with no singular definition—while acknowledging that this is often not how science is discussed or described by scientists or in discussion of science.

In our introduction, we explicitly state that there are many different approaches to formulating hypotheses, not all of which are deductive. However, we highlight that—despite this—deductivist approaches to generating and testing hypotheses are reified and viewed as superior and/or defining characteristics of science. In this way, the HDM is definitionally deductive, but does not represent all hypothesis-based approaches, and—in actual practice—it may be impossible to achieve "purely" deductivist processes. This does not change the fact that people have argued that this is the case, and that this sentiment is pervasive and carries influence in the actual practice of science.

To clarify this, we have greatly restructured the introduction to expand on our discussion of the existence of non-hypothesis-based approaches, non-deductive hypothesis-based methods exist, and moved our description of the HDM's inferential nature to come immediately after its first mention. We have also incorporated several additional references (e.g., Poincare, 1905; Glass & Hall, 2008) to clarify the historical grounds and development to current practice.

R2-2. Requested revision: Add an analytic subsection in the Results/Discussion that foregrounds domain- and purpose-specific interpretations; include a brief descriptive table (or SI) that lists each participant's sub-domain/purpose coding and inferred stance.

We appreciate this suggestion and spent considerable time exploring possible ways to do so. Ultimately, we believe that providing a concise table or list of participants' sub-domain would be inherently misleading or outright inaccurate. One of the first questions of the interview protocol asked participants to describe their research area or subdiscipline. The vast majority of participants did not give a "label" to their work, but rather provided an extended description of the types of projects they work on. Reproducing these extended descriptions would likely render participants identifiable within their communities. One participant explicitly stated that the intensively interdisciplinary nature of the work made it impossible to provide any label (or short collection of labels) that accurately described her field. Other participants provided a description of their work, and then explicitly stated several different labels they would use to identify their sub-domain, depending on contextual features such as what audience they were speaking to (and for what purpose). A handful of participants did provide concise labels for their field, but they were often too general (e.g., "inorganic materials chemistry") to be useful for this purpose.

However, we do agree that the manuscript would be improved by readers having a closer understanding of the types of sub-domains sampled in the data. Thus, we have added an aggregate description of sampled sub-domains, and justification for doing so (p. 7, clean copy of revised manuscript).

We also hesitate to infer a stance of each participants' purpose, on the basis that we did not ask a question directly targeting this—making the evidence this inference would be based on even more partial than the evidence for sub-discipline. Additionally, on the occasions where a participant does refer to their research purpose or aim, they often mention multiple simultaneous aims. For example, a personal purpose or aim to explore areas they are curious about, alongside an application-based aim to optimize solar cell efficiency or develop novel biomaterials. Thus, we do not believe it is possible with these data to accurately infer participants' purpose(s).

R2-3. Requested revision: Expand the Theoretical Foundations/Discussion with an evidence-indexed table or figure mapping CMNoS attributes to specific data excerpts and noting observed boundary conditions.

We hesitate to do this, as it blurs our results into our conceptual framework, and suggests our primary aim is theory-building to establish CMNoS as a grounded framework. It is true—and we do acknowledge in the manuscript—that the theoretical foundations employed in this analysis were influenced by factors the researchers noticed in preliminary analysis stages. However, after recognizing connections to both the nature of science and cultural master narrative literature, we used these literature bases to develop our CMNoS conceptual framework for this analysis. We note that our original manuscript included a discussion of the 5 key principles that define cultural master narratives, and how these principles can be seen reflected in the existing nature of science literature. In the revised

manuscript, we have expanded this discussion (pp 4-6, clean copy of revised manuscript), and included explicit reference to areas of our discussion that highlight the connections between our results and cultural master narrative principles (pp. 17-18, clean copy of revised manuscript).

We believe a systematic indexed map would suggest we are trying to use these data to advance this as a theoretical model tested by this study. Rather, by the nature of this study being an emergent realization, we believe these data are better positioned to raise the findings we discuss in the manuscript and to hopefully motivate further theory-building work that examines cultural master narratives about the nature of science.

R2-4. Requested revision: Provide a theme map and a definitions table; expand methodological notes on information power and reflexivity.

We have added a theme table (p. 10, clean copy of revised manuscript) and expanded the methods section, and include explicit discussion of how we managed power dynamics and reflexivity (see: Methods > Quality Considerations, pp. 8-9, clean copy of revised manuscript).

R2-5. Requested revision (one of the following): 1) Deposit de-identified excerpts sufficient to support claims in a reputable repository (e.g., OSF) with an access statement; or 2) Provide a controlled access mechanism (e.g., institutional data access/ethics committee contact, application procedure, and criteria) enabling qualified researchers to obtain data under safeguards; and 3) Ensure the revised Data Availability Statement reflects the chosen pathway and is internally consistent across the submission system and manuscript

We have added a section to the Supporting Information the includes excerpts of the core component of the interview transcripts that was analyzed in this study: participant responses to the question "Could you talk a bit about the role hypothesis testing plays in your work?". However, many participants recount detailed, highly specific examples from their prior or current projects, which could render them identifiable especially to members of their research communities. Thus, we have excluded some portions of these sections to protect participants' identity.

R2-6. Several sentences imply general claims about "science" writ large. To avoid overgeneralization, qualify claims to the sample and fields studied, reserving broader claims as conjectures for future work. Where you invoke system-level incentives (funding, publication norms), consider adding 1–2 citations from science-studies/science-policy to anchor these statements.

We have reviewed the full manuscript and made changes to be more precise about these terms, as well as our reference to "hypotheses" (writ large) vs specific approaches to

hypotheses (e.g, inductive hypotheses vs deductive hypotheses), which we think has greatly improved the manuscript.

W do note that in our discussion of NoS, we retain the reference to “science” (writ large), as these findings have been consistently found across many samples and fields. Similarly, we thus refer to the CMNoS in terms of science writ large, as that is supported by the NoS scholarship used to generate our formulation. Furthermore, we have retained the reference to “science” broadly in some places even where the citations may stem from one or a handful of subfields, because we refer to *those* publications making general, science-writ-large claims. Where we refer to funding and publication norms, we have also added new citations to our claims about funding and field norms.

R2-7. Add the incentive amount and form (gift card/transfer) in the Methods to the first paragraph that introduces the interview, to keep all participation details consolidated. Confirm whether participants could review their transcripts (member reflection) and, if not, state this explicitly.

In our submitted manuscript, the participant compensation information **is** included in the first paragraph that introduces the interview, which is the first paragraph in the Methods > Data Collection section. This is distinct from the first paragraph of the Methods section, which does not discuss interviews but does discuss ethics approval and participant consent. While we recognize that incentives/compensation are also relevant to considerations of study ethics, we believe it is particularly important to include in the broader description of the interviews to inform the readers’ evaluation of the nature of data collection.
