# Supplementary material for: “The hypothesis becomes forced”: Cultural narratives about deductive hypothesis testing influence scientists’ self-perception and actions
Source: PLoS One. 2026 Aug 21;21(8):e0346386. doi: 10.1371/journal.pone.0346386 (PMC13497266; doi:10.1371/journal.pone.0346386)
Supplement: Supplementary file 2 [file pone.0346386.s005.pdf]

We thank the editor and reviewer for their continued consideration of our original manuscript. Below, you can find the reviewer comments (in black), and our response (in blue). Throughout our response, line numbers correspond to the track-changes version of the revised manuscript.

Reviewer #3: I appreciate you pursuing such an interesting line of inquiry. It was a pleasure to review your work, and I found the content to be very engaging. This paper has already passed its first review. It's been improved a lot since the first version, and the methodology is now much more rigorous and solid. The paper has improved and become more sophisticated, however, I noticed a few points in the latest version that might a bit tricky for readers. Here are some suggestions to make it even better.

We thank the reviewer for their thoughtful attention to our work, their positive endorsement of its quality and importance, and their suggestions for further polishing this work.

Although the authors explicitly state in their theoretical foundations that they adopt a Critical Realism paradigm, there is a notable absence of an in-depth discussion utilizing its core ontology—the three stratified domains (the Empirical, the Actual, and the Real)—when interpreting the findings. The significance of the five themes derived through Reflexive Thematic Analysis (RTA) in this study becomes much more multidimensional and profound when interpreted through this three-tiered structure of Critical Realism. For instance, the anxiety and identity threats experienced by participants (Theme 4) can be mapped to the observable 'domain of the empirical'; the exploratory research and HARKing practices occurring in the field (Themes 2, 3, and 5) to the 'domain of the actual'; and the cultural master narrative of science (CMNOS, Theme 1), which acts as the underlying structural force continuously generating these experiences and practices, to the 'domain of the real'. Therefore, building upon the stated paradigm, it would significantly strengthen the paper if the authors could expand the Discussion section by deeply reinterpreting the inductively derived RTA themes through the lens of Critical Realism's three-tiered structure. Furthermore, presenting a visualized conceptual model that clearly illustrates these multi-layered interactions would significantly strengthen the study's methodological congruence and theoretical completeness. […]my point is that the data and themes derived through RTA need to be explained and interpreted within the theoretical framework of CR. Since the authors explicitly stated that they adopted a Critical Realist perspective, it seems necessary to address this connection.

We appreciate the reviewer raising this point, and have realized that our phrasing ("employing a critical realist and contextualist paradigm") was unclear: in this work, we drew on critical realism as an epistemology, but did not extend our consideration of critical realism to using its ontological framework as an analytical tool.

We appreciate the reviewer's thoughtful insight about how our results triangulate with the ontological principles of critical realism, and agree that our findings do overlap with these ontological principles in this way. However, as we did not conduct this work with this ontology of the empirical, actual, and real in mind, we do not believe it is necessary to address this connection. Instead, we have clarified that we employed a critical realist *epistemology* to structure this project's research design, with illustrative examples of how that informed framework selection (lines 108-120). While a retrospective triangulation with critical realism's ontological principles could still be made as a way to establish trustworthiness of the findings, we believe the current methods used to establish trustworthiness of the findings (see: Methods > Quality Considerations) are sufficient, and we highlight that reviewers have raised no concerns about

how trustworthiness criteria were addressed. Given this, we are concerned that tacking on a retrospective triangulation with this ontological framework would provide “more of the same” in terms of establishing trustworthiness—but, since it relies on having presented the results, incorporating this discussion would require additional description of trustworthiness in the discussion and results section, which we are concerned would distract the target audience from our key points.

1. The author states on page 4 that “it is important to recognize that, regardless of the NoS formulation employed, implicitly-developed views about science often conflict with established tenets of NoS”.
From the perspective of the Family Resemblance Approach (FRA), the participants’ responses could interpreted not as ‘conflicts’ with a master narrative, but as legitimate disciplinary variations reflecting ‘family resemblances’ across different scientific fields or theme. By framing these nuances simply as ‘conflicts’ regardless of the formulation used, the study risks oversimplifying the phenomena.

We appreciate the reviewer raising this point, as it highlights a need for better clarity. The FRA provides greater flexibility for disciplinary variations than other formulations, but this flexibility still has limits. In other words: the FRA acknowledges that some partial or narrow formulations of science may accurately reflect the nature of science in specific contexts. However, this does not mean that all potential views of science are valid reflections of reality. For example: it would conflict with the FRA to generalize context-specific partial/narrow NoS formulations to describe science in general beyond the context in question. Similarly, the CV, FRA, and revised FRA all acknowledge that, because science is conducted by humans, it is not possible for science to be purely objective, free of bias, and/or culturally neutral. However, as seen in the literature, the conception of science as objective and culturally neutral is very widespread and pervasive.

We have revised the language in lines 143-152 to better clarify this in the manuscript.

The theoretical coherence of the manuscript could be further enhanced by refining the framing as follows:

> ① Minimize or delete unnecessary references to alternative perspectives like FRA or FoS if they are not actively used as the primary analytical lens.
>
> In principle, to enable the reader to evaluate the limitations of our work we believe it is important to acknowledge any substantial the critiques frameworks we employ, even if those critiques are not used as an analytical lens. Additionally, in this study we use both the CV NoS and the revised FRA NoS to operationalize our conception of the reality of the nature of science. We have made substantial changes (see: Lines 143-171 & Figure 1) to better communicate how, for our purposes, both models are mutually compatible and complementary.
>
> ② Remove the phrase “regardless of the NoS formulation employed”, as it creates a contradiction when the analysis subsequently relies on specific tenets.
>
> We have removed this phrasing.

③ Instead, explicitly state that this study adopts the Consensus View (CV) as its primary analytical framework. (I have observed that the authors are relying exclusively on the tenets of CV (Consensus View) in their interpretation of the findings. It was for this reason that I put forward the aforementioned suggestions.)

We have clarified the role of CV NoS as a primary theoretical framework, and also clarified how we use the revised FRA to supplement this theory in alignment with our critical realist epistemology (see lines 162-171). To further clarity our use of this framework (as more of a tool for operationalization than for analysis), we expand on how we situate these frameworks relative to the conceptual framework developed to describe the CMNoS (see lines 249-263, Figure 2).

2. Additionally, in the same sentence, it is logically unclear why implicitly-acquired views must be compared specifically against the CV, "regardless of the Nos formulation employed." If one adopts an alternative formulation such as the FRA, a deviation from the CV's tenets is not a 'conflict' to be corrected, but a legitimate epistemological variation. By insisting on a comparison with 'established tenets (which function here as the CV)", the author inadvertently privileges the CV over other formulations.

We believe our response to the above points above also address this point.

3. The author presents a pivotal theoretical argument in lines 690-692: "This is well aligned with the NoS, which describes science as a creative and social process. However, this is not conserved in the CMNoS, which associates "good" science with being strictly structured and objective." While this conclusion is central to the study, I find a significant structural issue regarding its substantiation.

The empirical foundation for this claim—specifically regarding the prioritization of intuition over formal hypotheses—is located several pages earlier in the Results section (near lines 525–526). By failing to provide a summarized re-citation or an explicit cross-reference within the Discussion section, the author forces the reader to rely solely on memory or engage in the inconvenient task of constantly flipping back and forth between pages to verify the argument.

The structural that strictly segregates the 'results', discussion', and 'implications' poses significant challenges for the reader. If this structure is maintained, the discussion would greatly benefit from explicit cross-referencing to the specific portions of the results that support each statement during the narrative of the discussion and implications. A lack of immediate connectivity between the participants' narratives and the theoretical analysis may cause the author's logic to be perceived as an 'interpretive leap', which could potentially obscure the methodological rigor that the authors have achieved.

We would be very concerned if the substantiation of the CMNoS were lacking. However, we are relieved to see that the reviewer recognizes the empirical foundation for this claim in the results section, and thus understand this to be an issue of organization of the communicated work rather

than an issue of insufficient substantiation or evidence. We believe changes made in response to comment 1.3 have also assisted in addressing this point.

Furthermore, as a discipline-based education research (DBER) study, this work operates within scientific disciplines with strong norms surrounding the results / discussion / implication structure in peer-reviewed publications. For this reason, we strongly prefer to retain the results / discussion / implication structure, and regret that this structure creates difficulties for readers in adjacent disciplines where this may not be the norm.

We note that, as written, the discussion section already includes significant reference to earlier sections, and the bolded theme descriptions map directly to subsection titles in the results to make it easier for the reader to cross-reference. To address the specific portion indicated by the reviewer, we have added an analogous reference to the relevant portion of the results (highlight added for emphasis):

> "Participants' perceptions of themselves influences their work, as reflected in the theme **Personal feelings and inclinations influence participants' work**. This theme reflects how emotions and personal dispositions shape participants' work. For example, Iris and Zayn describe how their enthusiasm and curiosity informs their research directions, and Henri describes a good sense of intuition as essential for his work. This is well aligned with the NoS, which describes science as a creative and social process. However, this is not conserved in the CMNoS, which associates "good" science with being strictly structured and objective." (lines 735-741)

4. The author provides an extensive review of the Consensus View (CV) of the nature of science (NOS) in chapter 2, establishing it as a primary analytical framework. However, there is a significant lack of clarity regarding how this framework is practically applied to the data and how it interacts with the newly proposed CMNoS.

> ① The CV consists of 7 to 8 distinct tenets (e.g., subjectivity, creativity, social-cultural embeddedness). The CMNoS, as described in this study, appears to focus almost exclusively on the 'myth of a single scientific method' (specifically, the hypothetico-deductive model). This raises a critical question: Is CMNoS merely a 'misconception' of a single CV tenet regarding the diversity of scientific methods, or it a truly a 'master narrative' that encompasses the entire NOS? If it is the former, it would be helpful to elaborate on why it is treated as a grand cultural narrative rather than a localized misconception within the NOS framework.
>
> The CMNoS is a conceptual framework developed for this study, distinct from the CV. We follow Luft et al. 2022's description of conceptual frameworks: a framework developed by the researcher (informed by the literature, experience, preliminary analyses of data, etc.) for the purpose of communicating researcher's conceptualization of topics and relationships relevant to the interpretation of data within the context of the study. In formulating a conceptual framework, not all components of related theoretical frameworks may be relevant. Cultural master narratives exert influence over how individuals perceive things to be, but they do not necessarily accurately reflect reality—thus, components of CV may not be present in the data.

We do not argue the CMNoS is a complete or accurate way to conceptualize the reality of science. Rather, informed by the NoS scholarship, we argue that it is a widespread narrative about science held strongly by scientists and non-scientists alike. As we here employ the CV and RFA models of NoS to inform our thinking about science, we would argue that the CMNoS' implicit assumption that the hypothetico-deductive model should generalize to describe the process of science in all disciplines, and the neglect of other approaches to or contributors to the nature of science (e.g., creativity, sociocultural systems, etc.) is a misconception and overgeneralization of a single tenant of the NoS. In other words: the CMNoS conflates all of science with one narrow and partial view of science.

To better communicate this in the manuscript, we have expanded our discussion of the CMNoS and incorporated an illustrative conceptual map (see lines 242-263 & Figure 2).

② In the discussion, the author introduces other tenets such as 'imagination and creativity' to critique the rigidity of the CMNoS. This suggests that the analysis is not limited to 'methodological diversity' but involves multiple CV tenets. The author needs to explicitly specify which of the 7-8 tenets are being utilized as analytical lenses and which are excluded (and why).

I regret we are unsure what the reviewer means in this comment. In the discussion section, the only mention of rigidity is unrelated to imagination and creativity. However, we believe that clarity around this point is likely provided by changes made in the theoretical foundations section in response to the above comments.

Furthermore, we reiterate that we are not using CV as a strict analytical lens but instead use it—alongside the revised family resemble approach—as a theoretical framework to operationalize the reality of the nature of science. Against this backdrop, we develop a conceptual framework to structure and operationalize our definition of cultural master narratives of science. Together, these three frameworks are concurrently used to inform our data analysis and interpretations, and we draw on pieces of each as we perceive them to be relevant to the data. We find the CMNoS to be highly productive in understanding the perceptions communicated by the participants, and we find the CV and revised FRA productive in understanding how and why the CMNoS does not accurately reflect reality. We believe the revisions made to the prior comments have also better clarified this in the manuscript.

③ I have a fundamental concern regarding whether the CMNoS, as currently presented, a possesses the functional properties of a rigorous analytical framework or if it is merely a single-sentence definition used as a general guideline during the reflexive thematic analysis. If the CMNoS serves only as a broad interpretive lens rather than a structured system for data categorization, the current usage of the term 'framework' might lead to reader expectations regarding methodological structure that are not fully realized in the text. Providing a conceptual map could resolve this. To justify the use of this term, the author should make a concerted effort to visualize the CMNoS, providing a structural map or a model that clarifies its internal dimensions and how it systematically interacts with the raw data, rather than leaving it as an ambiguous conceptual label.

We believe changes made to address the preceding points have also address this, including the addition of a conceptual map to model the CMNoS (Figure 2).

5. I believe the authors' choice to adopt Reflexive Thematic Analysis (RTA) to answer their research questions is a highly appropriate methodological decision. My critique by no means implies forcing the Nature of Science (NOS) to be used as a rigid "deductive codebook" at the expense of undermining RTA's flexible epistemological principles. While RTA is undoubtedly an excellent methodology for reading the broader context of the data and emergently weaving together meanings, the analytical step of identifying or mapping that "this participant's utterance demonstrates a specific NOS perspective" typically necessitates a clear analytical baseline to ground such interpretive judgments transparently.
In other words, to determine that a specific NOS tenet has manifested in the raw data—which serves as the foundational material for deriving overarching themes in RTA—there must be an antecedent operational definition of the criteria the researchers used to examine the data. Therefore, to prevent arbitrariness in qualitative interpretation and to ensure analytical transparency and rigor, I strongly recommend that the authors explicitly detail in the Data Analysis section the specific standards they utilized when identifying and mapping particular Consensus View (CV) tenets within the data.

We appreciate this point, but believe this comment stems from misunderstanding the role of NoS and CMNoS frameworks used in this study. Thus, we believe this point has been addressed in our revisions and response made to address the several of the preceding points. In summary: we are not identifying and mapping participants' utterances to CV tenets. Rather, as we communicate in the manuscript, informed by our preliminary analysis of the data, we returned to the literature and developed a conceptual framework (CMNoS) to help us understand why, when asked about their use of hypothesis testing, participants responded by making broad statements about the nature of science writ large. Thus, while we remained attentive to the possibility of CV tenets manifesting in the data, we primarily notice instances of the CMNoS manifesting in the data, and draw on the scholarship of cultural master narratives to discuss implications of this for participants' self-perception and identity as scientists.
