# Supplementary material for: “The hypothesis becomes forced”: Cultural narratives about deductive hypothesis testing influence scientists’ self-perception and actions
Source: PLoS One. 2026 Aug 21;21(8):e0346386. doi: 10.1371/journal.pone.0346386 (PMC13497266; doi:10.1371/journal.pone.0346386)
Supplement: Supplementary file 3 [file pone.0346386.s002.docx]

I appreciate you pursuing such an interesting line of inquiry. It was a pleasure to review your work, and I found the content to be very engaging.

This paper has already passed its first review. It’s been improved a lot since the first version, and the methodology is now much more rigorous and solid.

The paper has improved and become more sophisticated, however, I noticed a few points in the latest version that might a bit tricky for readers. Here are some suggestions to make it even better.

[Although the authors explicitly state in their theoretical foundations that they adopt a Critical Realism paradigm, there is a notable absence of an in-depth discussion utilizing its core ontology—the three stratified domains (the Empirical, the Actual, and the Real)—when interpreting the findings. The significance of the five themes derived through Reflexive Thematic Analysis (RTA) in this study becomes much more multidimensional and profound when interpreted through this three-tiered structure of Critical Realism. For instance, the anxiety and identity threats experienced by participants (Theme 4) can be mapped to the observable 'domain of the empirical'; the exploratory research and HARKing practices occurring in the field (Themes 2, 3, and 5) to the 'domain of the actual'; and the cultural master narrative of science (CMNOS, Theme 1), which acts as the underlying structural force continuously generating these experiences and practices, to the 'domain of the real'. Therefore, building upon the stated paradigm, it would significantly strengthen the paper if the authors could expand the Discussion section by deeply reinterpreting the inductively derived RTA themes through the lens of Critical Realism's three-tiered structure. Furthermore, presenting a visualized conceptual model that clearly illustrates these multi-layered interactions would significantly strengthen the study's methodological congruence and theoretical completeness.]

I am not suggesting that Critical Realism (CR) should be used as a codebook for Reflexive Thematic Analysis (RTA). Rather, my point is that the data and themes derived through RTA need to be explained and interpreted within the theoretical framework of CR. Since the authors explicitly stated that they adopted a Critical Realist perspective, it seems necessary to address this connection.

1. The author states on page 4 that “it is important to recognize that, regardless of the NoS formulation employed, implicitly-developed views about science often conflict with established tenets of NoS”.

From the perspective of the Family Resemblance Approach (FRA), the participants’ responses could interpreted not as ‘conflicts’ with a master narrative, but as legitimate disciplinary variations reflecting ‘family resemblances’ across different scientific fields or theme. By framing these nuances simply as ‘conflicts’ regardless of the formulation used, the study risks oversimplifying the phenomena.

The theoretical coherence of the manuscript could be further enhanced by refining the framing as follows:

① Minimize or delete unnecessary references to alternative perspectives like FRA or FoS if they are not actively used as the primary analytical lens.

② Remove the phrase “regardless of the NoS formulation employed”, as it creates a contradiction when the analysis subsequently relies on specific tenets.

③ Instead, explicitly state that this study adopts the Consensus View (CV) as its primary analytical framework. (I have observed that the authors are relying exclusively on the tenets of CV (Consensus View) in their interpretation of the findings. It was for this reason that I put forward the aforementioned suggestions.)

2. Additionally, in the same sentence, it is logically unclear why implicitly-acquired views must be compared specifically against the CV, “regardless of the Nos formulation employed.” If one adopts an alternative formulation such as the FRA, a deviation from the CV’s tenets is not a ‘conflict’ to be corrected, but a legitimate epistemological variation. By insisting on a comparison with ‘established tenets (which function here as the CV)”, the author inadvertently privileges the CV over other formulations.

3. The author presents a pivotal theoretical argument in lines 690-692: “This is well aligned with the NoS, which describes science as a creative and social process. However, this is not conserved in the CMNoS, which associates “good” science with being strictly structured and objective.” While this conclusion is central to the study, I find a significant structural issue regarding its substantiation.

The empirical foundation for this claim—specifically regarding the prioritization of intuition over formal hypotheses—is located several pages earlier in the Results section (near lines 525–526). By failing to provide a summarized re-citation or an explicit cross-reference within the Discussion section, the author forces the reader to rely solely on memory or engage in the inconvenient task of constantly flipping back and forth between pages to verify the argument.

The structural that strictly segregates the ‘results’, discussion’, and ‘implications’ poses significant challenges for the reader. If this structure is maintained, the discussion would greatly benefit from explicit cross-referencing to the specific portions of the results that support each statement during the narrative of the discussion and implications. A lack of immediate connectivity between the participants’ narratives and the theoretical analysis may cause the author’s logic to be perceived as an ‘interpretive leap’, which could potentially obscure the methodological rigor that the authors have achieved.

4. The author provides an extensive review of the Consensus View (CV) of the nature of science (NOS) in chapter 2, establishing it as a primary analytical framework. However, there is a significant lack of clarity regarding how this framework is practically applied to the data and how it interacts with the newly proposed CMNoS.

① The CV consists of 7 to 8 distinct tenets (e.g., subjectivity, creativity, social-cultural embeddedness). The CMNoS, as described in this study, appears to focus almost exclusively on the ‘myth of a single scientific method’ (specifically, the hypothetico-deductive model). This raises a critical question: Is CMNoS merely a ‘misconception’ of a single CV tenet regarding the diversity of scientific methods, or it a truly a ‘master narrative’ that encompasses the entire NOS? If it is the former, it would be helpful to elaborate on why it is treated as a grand cultural narrative rather than a localized misconception within the NOS framework.

② In the discussion, the author introduces other tenets such as ‘imagination and creativity’ to critique the rigidity of the CMNoS. This suggests that the analysis is not limited to ‘methodological diversity’ but involves multiple CV tenets. The author needs to explicitly specify which of the 7-8 tenets are being utilized as analytical lenses and which are excluded (and why).

③ I have a fundamental concern regarding whether the CMNoS, as currently presented, a possesses the functional properties of a rigorous analytical framework or if it is merely a single-sentence definition used as a general guideline during the reflexive thematic analysis. If the CMNoS serves only as a broad interpretive lens rather than a structured system for data categorization, the current usage of the term 'framework' might lead to reader expectations regarding methodological structure that are not fully realized in the text. Providing a conceptual map could resolve this. To justify the use of this term, the author should make a concerted effort to visualize the CMNoS, providing a structural map or a model that clarifies its internal dimensions and how it systematically interacts with the raw data, rather than leaving it as an ambiguous conceptual label.

5. I believe the authors' choice to adopt Reflexive Thematic Analysis (RTA) to answer their research questions is a highly appropriate methodological decision. My critique by no means implies forcing the Nature of Science (NOS) to be used as a rigid "deductive codebook" at the expense of undermining RTA's flexible epistemological principles. While RTA is undoubtedly an excellent methodology for reading the broader context of the data and emergently weaving together meanings, the analytical step of identifying or mapping that "this participant's utterance demonstrates a specific NOS perspective" typically necessitates a clear analytical baseline to ground such interpretive judgments transparently.

In other words, to determine that a specific NOS tenet has manifested in the raw data—which serves as the foundational material for deriving overarching themes in RTA—there must be an antecedent operational definition of the criteria the researchers used to examine the data. Therefore, to prevent arbitrariness in qualitative interpretation and to ensure analytical transparency and rigor, I strongly recommend that the authors explicitly detail in the Data Analysis section the specific standards they utilized when identifying and mapping particular Consensus View (CV) tenets within the data.
