# Supplementary material for: “The hypothesis becomes forced”: Cultural narratives about deductive hypothesis testing influence scientists’ self-perception and actions
Source: PLoS One. 2026 Aug 21;21(8):e0346386. doi: 10.1371/journal.pone.0346386 (PMC13497266; doi:10.1371/journal.pone.0346386)
Supplement: Supplementary file 4 [file pone.0346386.s004.docx]

I appreciate the authors’ careful and substantial revision of the manuscript. The second-round changes have improved the clarity of the argument, the consistency of the theoretical framing, and the transparency of the methods.

The authors have also responded directly to the main theoretical and structural issues raised in the previous review.
The addition of Figure 2 now presents the conceptual framework of the Cultural Master Narrative of Science (CMNOS) in a clear visual form, making its dimensions and sequence of processes easier to follow.
The updated Theoretical Foundations section now treats the Consensus View (CV) as the explicit reference point, and integrates the revised Family Resemblance Approach (FRA) without the earlier ambiguity around “conflict” wording.
In the Discussion, links to specific participant narratives and themes from the Results are now explicitly signposted, which strengthens the connection between the empirical analysis and the theoretical claims.

More broadly, the study offers a useful contribution to discipline-based science education and higher education research. It shows how persistent cultural master narratives about hypothetico–deductive methods shape postdoctoral researchers’ self-understanding, research practices, and scientific communication. In light of the strengthened argumentation and methodological clarity in this version, I recommend the manuscript for publication in PLOS ONE.
