# Supplementary material for: “The hypothesis becomes forced”: Cultural narratives about deductive hypothesis testing influence scientists’ self-perception and actions
Source: PLoS One. 2026 Aug 21;21(8):e0346386. doi: 10.1371/journal.pone.0346386 (PMC13497266; doi:10.1371/journal.pone.0346386)
Supplement: Peer review history

# Peer review: Decision Letter 0

**DOI:** 10.1371/journal.pone.0346386.r001

**Authors:** Nadia Rehman

26 Sep 2025

Dear Dr. James,

Thank you for submitting your manuscript to PLOS ONE. After careful consideration, we feel that it has merit but does not fully meet PLOS ONE’s publication criteria as it currently stands. Therefore, we invite you to submit a revised version of the manuscript that addresses the points raised during the review process.

==============================

This manuscript presents a qualitative study exploring postdoctoral researchers’ perspectives on hypothesis-driven methods (HDM) and their influence on scientific practice and identity. The paper makes a potentially valuable contribution by extending Nature-of-Science (NoS) scholarship into the postdoctoral context and by framing perceptions of HDM as a cultural master narrative. The rationale and research gap are clearly articulated, and the discussion is aligned with the stated research questions.

However, the manuscript requires substantial clarification and expansion of its **methodological reporting**  to ensure transparency, trustworthiness, and compliance with qualitative reporting standards (e.g., COREQ). While the thematic analysis is appropriate, key details about sampling, data collection, and analysis procedures are missing or insufficiently specified. Addressing these points is essential before the manuscript can be considered further.

==============================

Please submit your revised manuscript by Nov 10 2025 11:59PM. If you will need more time than this to complete your revisions, please reply to this message or contact the journal office at plosone@plos.org. When you're ready to submit your revision, log on to https://www.editorialmanager.com/pone/ and select the 'Submissions Needing Revision' folder to locate your manuscript file.

- A rebuttal letter that responds to each point raised by the academic editor and reviewer(s). You should upload this letter as a separate file labeled 'Response to Reviewers'.
- A marked-up copy of your manuscript that highlights changes made to the original version. You should upload this as a separate file labeled 'Revised Manuscript with Track Changes'.
- An unmarked version of your revised paper without tracked changes. You should upload this as a separate file labeled 'Manuscript'.

If you would like to make changes to your financial disclosure, please include your updated statement in your cover letter. Guidelines for resubmitting your figure files are available below the reviewer comments at the end of this letter.

If applicable, we recommend that you deposit your laboratory protocols in protocols.io to enhance the reproducibility of your results. Protocols.io assigns your protocol its own identifier (DOI) so that it can be cited independently in the future. For instructions see: https://journals.plos.org/plosone/s/submission-guidelines#loc-laboratory-protocols. Additionally, PLOS ONE offers an option for publishing peer-reviewed Lab Protocol articles, which describe protocols hosted on protocols.io. Read more information on sharing protocols at https://plos.org/protocols?utm_medium=editorial-email&utm_source=authorletters&utm_campaign=protocols.

We look forward to receiving your revised manuscript.

Kind regards,

Nadia Rehman, Ph.D.

Academic Editor

PLOS ONE

Journal Requirements:

When submitting your revision, we need you to address these additional requirements.

1. Please ensure that your manuscript meets PLOS ONE’s style requirements, including those for file naming. The PLOS ONE style templates can be found at

https://journals.plos.org/plosone/s/file?id=wjVg/PLOSOne_formatting_sample_main_body.pdf and

https://journals.plos.org/plosone/s/file?id=ba62/PLOSOne_formatting_sample_title_authors_affiliations.pdf

2. Thank you for stating the following financial disclosure:

“This material is based upon work supported by the National Science Foundation under

Award No. 2320454”

Please state what role the funders took in the study. If the funders had no role, please state: "The funders had no role in study design, data collection and analysis, decision to publish, or preparation of the manuscript."

If this statement is not correct you must amend it as needed.

Please include this amended Role of Funder statement in your cover letter; we will change the online submission form on your behalf.

3. In the online submission form, you indicated that “The data analyzed in this work are transcripts of post-doctoral scholars' description of their work and the concepts and skills used in their work. Thus, open sharing of these data could render some participants identifiable. Upon request, the corresponding author will share all necessary portions of de-identified transcripts.”

All PLOS journals now require all data underlying the findings described in their manuscript to be freely available to other researchers, either 1. In a public repository, 2. Within the manuscript itself, or 3. Uploaded as supplementary information.

This policy applies to all data except where public deposition would breach compliance with the protocol approved by your research ethics board. If your data cannot be made publicly available for ethical or legal reasons (e.g., public availability would compromise patient privacy), please explain your reasons on resubmission and your exemption request will be escalated for approval.

4. If the reviewer comments include a recommendation to cite specific previously published works, please review and evaluate these publications to determine whether they are relevant and should be cited. There is no requirement to cite these works unless the editor has indicated otherwise.

5. We note that there is identifying data in the Table 1. Due to the inclusion of these potentially identifying data, prior to sharing human research participant data, authors should consult with an ethics committee to ensure data are shared in accordance with participant consent and all applicable local laws.

Data sharing should never compromise participant privacy. It is therefore not appropriate to publicly share personally identifiable data on human research participants. The following are examples of data that should not be shared:

-Name, initials, physical address

-Ages more specific than whole numbers

-Internet protocol (IP) address

-Specific dates (birth dates, death dates, examination dates, etc.)

-Contact information such as phone number or email address

-Location data

-ID numbers that seem specific (long numbers, include initials, titled “Hospital ID”) rather than random (small numbers in numerical order)

Data that are not directly identifying may also be inappropriate to share, as in combination they can become identifying. For example, data collected from a small group of participants, vulnerable populations, or private groups should not be shared if they involve indirect identifiers (such as sex, ethnicity, location, etc.) that may risk the identification of study participants.

Additional guidance on preparing raw data for publication can be found in our Data Policy (https://journals.plos.org/plosone/s/data-availability#loc-human-research-participant-data-and-other-sensitive-data) and in the following article: http://www.bmj.com/content/340/bmj.c181.long.

Please remove or anonymize all personal information (Name), ensure that the data shared are in accordance with participant consent, and re-upload a fully anonymized data set.

**Additional Editor Comments:**

****Standardize terminology, clarify analytic stance, and expand description of coding and theme development.

****Provide a clear Sampling & Recruitment subsection, including rationale for sample size.

****Specify interview language, transcription workflow, and interviewer roles. Add a reflexive note on power dynamics.

****Clarify stance on member checking, state if software was used, and include a theme table (main text or supplementary).

****A COREQ checklist should be completed and uploaded to ensure full methodological transparency.

****The reference list appropriately includes foundational sources but relies heavily on older literature (>5 years). Recent publications (2020–2025) are included but could be strengthened further by incorporating the latest scholarship on:

Qualitative reporting standards in higher education research. (for Qualitative reporting follow these studies and cite them; https://doi.org/10.1186/s12909-025-07185-9 https://doi.org/10.1155/2024/1271802 )

Researcher identity and methodological culture in STEM postdocs.

Current debates around hypothesis testing, openness, and HARKing.

****Ensure consistent abbreviations (e.g., HDM vs HDMs)

**** Consider adding a visual model illustrating (if possible)

[Note: HTML markup is below. Please do not edit.]

Reviewers' comments:

Reviewer’s Responses to Questions

**Comments to the Author**

1. Is the manuscript technically sound, and do the data support the conclusions?

Reviewer #1: Partly

Reviewer #2: Yes

**********

2. Has the statistical analysis been performed appropriately and rigorously?

Reviewer #1: N/A

Reviewer #2: N/A

**********

3. Have the authors made all data underlying the findings in their manuscript fully available?

The PLOS Data policy

Reviewer #1: Yes

Reviewer #2: No

**********

4. Is the manuscript presented in an intelligible fashion and written in standard English?

Reviewer #1: Yes

Reviewer #2: Yes

**********

Reviewer #1: This paper presents a distinctive study that analyzes scientists’ self-perceptions from the perspective of the nature of science. Overall, the paper is interesting, but there are some areas that would benefit from further clarification and improvement.

Line 71

It would be helpful if the authors could provide a clearer rationale for focusing on postdoctoral researchers in materials and chemistry. In particular, connecting this choice to specific aspects of the nature of science, or to the unique modes of thinking, paradigms, and experimental approaches in materials and chemistry, would enhance the contribution of the study.

Lines 91 and 562

The research questions are clearly stated; however, in the Discussion section, I recommend adding concise answers to each research question. It may also strengthen the clarity of the discussion if the subheadings correspond explicitly to these research questions.

Line 224

The description of the interview content could be elaborated further. Please specify what additional questions were asked beyond those already mentioned, so that the reader can better understand the scope of the interviews.

Line 520

Table 1 presents information on Experience and Prior Training. Were there no noteworthy patterns or features related to these variables? If none were observed, a brief statement clarifying this would improve the completeness of the discussion.

Reviewer #2: <general comments=" ">

This emergent qualitative study analyzes 16 semi‑structured interviews with postdoctoral scholars in materials/chemistry–related fields to examine how cultural master narratives about science (CMNs) shape perceptions of the hypothetico‑deductive method (HDM) and, in turn, scientists’ self‑perception and practice. Through reflexive thematic analysis, the manuscript argues that a deductive, hypothesis‑testing ideal is perceived as the gold standard, yet participants frequently describe non‑HDM practices as more productive in their day‑to‑day work, creating dissonance that must be managed via alternative narratives. The topic is timely and raises important questions for science education and research training.

While this paper makes valuable contributions, I propose several major revisions that I believe would significantly strengthen its theoretical foundation and interpretive depth. My recommendations are outlined below.

________________________________________

<major comments=" ">

1. Theoretical positioning of the Hypothetico–Deductive Method (HDM)

The Introduction currently frames HDM within “deductivist approaches” and treats it as the paradigmatic (“gold standard”) model of scientific inquiry. Please clarify the inferential architecture of HDM and justify its placement under a purely deductive banner.

- Clarify components and logic: HDM, as widely construed in contemporary philosophy of science and science‑education discourse, involves a composite workflow: (i) hypothesis generation that often draws on inductive and/or abductive (Peircean) reasoning; (ii) deductive derivation of testable consequences; and (iii) inductive/abductive appraisal of evidence relative to predictions. The manuscript would benefit from an explicit account of these phases and of why you classify HDM as “deductive,” given this mixed‑mode character. If you retain a deductivist classification, please present principled criteria for that choice and acknowledge alternative taxonomies used in the literature.

- Historicize the rise of HDM as a master narrative: To support your “cultural master narrative of science” (CMNoS) claims, please add a concise historical sketch: (a) early‑modern ambivalence toward hypotheses (e.g., Newton’s anti‑hypothetical rhetoric vs. Continental defenses of theoretical speculation); (b) late‑19th‑century normalization of unobservable theoretical entities (e.g., atomism) and model‑based explanation; (c) 20th‑century codifications (e.g., Popperian falsificationism) that elevated hypothesis‑testing as a professional ideal; and (d) the role of scientist training and research evaluation (funding, publication norms) in stabilizing HDM as a cultural master narrative. This historical scaffolding is essential for readers to understand why HDM became dominant and how it acquires the “ubiquity, invisibility, utility, rigidity, and compulsory” properties you analyze under CMNoS.

- Terminological precision: Distinguish “hypothesis‑testing” from “hypothesis‑driven” research; and clarify “abduction” (inference to the best explanation) vs. induction, since participants’ narratives appear to straddle exploratory (data‑driven) and explanatory (model‑based) logics.

Requested revision: Insert a dedicated subsection (in Introduction or Theoretical Foundations) that (i) delineates HDM’s mixed inferential structure, (ii) justifies your deductivist labeling, and (iii) offers a brief historically grounded rationale for HDM’s dominance and linkage to CMNoS.

________________________________________

2. Interpreting accounts through domain and purpose specificity

Participants span varied sub‑domains (e.g., synthesis, characterization, computational/theory, device design) and heterogeneous research purposes (e.g., discovery‑oriented vs. design/engineering vs. measurement/instrumentation). These factors plausibly mediate the fit and value of hypothesis‑driven approaches. To deepen explanatory power:

- Map domain/purpose to stances: Provide a cross‑tab (or matrix in text) that links each participant’s sub‑domain and predominant research purpose to their stance toward HDM (e.g., embrace, qualified embrace, limited fit, reframing via alternative narratives). Use 1–2 exemplar quotes per cell where feasible.

- Interpretive payoffs: Discuss why applied/design or tool‑building contexts may differentially privilege iterative prototyping, parameter search, or heuristic exploration (which your data already hint at), whereas theory‑driven contexts may align more closely with deductive testing. Consider how institutional context (public/private R1 vs. gov’t labs) and evaluation pressures (funding, publication venues) reinforce particular narratives.

- Scope and transferability: Explicitly delimit generalization to postdoctoral scholars in materials/chemistry‑related areas; propose how future work could test whether similar CMNoS dynamics appear in, e.g., ecology, clinical sciences, or high‑energy physics.

Requested revision: Add an analytic subsection in the Results/Discussion that foregrounds domain‑ and purpose‑specific interpretations; include a brief descriptive table (or SI) that lists each participant’s sub‑domain/purpose coding and inferred stance.

________________________________________

3. CMN → CMNoS: conceptual sharpness and empirical warrant

Your adoption of cultural master narratives is thoughtful. To further strengthen the theoretical contribution:

- Differentiate CMN vs. CMNoS: Make explicit how CMNoS operationalizes the five CMN attributes (ubiquity, invisibility, utility, rigidity, compulsory nature) within scientific work cultures. Show where the data instantiate each attribute (linking to quotes), and where they do not—to avoid reifying a single monolithic narrative.

- Alternative or competing narratives: Briefly acknowledge other live narratives (e.g., exploratory data analysis/Big Data discovery, model‑based mechanistic explanation, engineering optimization) and indicate how they intersect or compete with an HDM‑centric narrative in your sample.

Requested revision: Expand the Theoretical Foundations/Discussion with an evidence‑indexed table or figure mapping CMNoS attributes to specific data excerpts and noting observed boundary conditions.

________________________________________

4. Methodological transparency in reflexive thematic analysis (RTA)

The manuscript already describes memoing, collaborative theme generation, and credibility strategies. For full transparency and reproducibility within RTA:

- Theme development artifacts: Provide a concise theme map and a table of final themes with analytic definitions, inclusion/exclusion criteria, and exemplar quotes. If space is tight, include as Supporting Information.

- Analytic adequacy: Clarify how you judged adequacy of information power/coverage for the focal question (not “saturation” per se in RTA), and how disconfirming evidence checks influenced theme boundaries.

- Researcher reflexivity: You note interviewer roles and reflexivity. Please expand with a brief positionality paragraph for all analysts and how these positions were used as analytic resources rather than sources of bias.

Requested revision: Provide a theme map and a definitions table; expand methodological notes on information power and reflexivity.

________________________________________

5. Data availability and compliance with PLOS ONE policy

Your current Data Availability Statement indicates that de‑identified transcript portions will be shared upon request because of identifiability concerns. PLOS ONE policy does not accept “available on request” as sufficient; exceptions require a clear access pathway (e.g., controlled‑access repository or institutional data access committee) and justification.

Requested revision (one of the following): 1) Deposit de‑identified excerpts sufficient to support claims in a reputable repository (e.g., OSF) with an access statement; or 2) Provide a controlled access mechanism (e.g., institutional data access/ethics committee contact, application procedure, and criteria) enabling qualified researchers to obtain data under safeguards; and 3) Ensure the revised Data Availability Statement reflects the chosen pathway and is internally consistent across the submission system and manuscript.

________________________________________

6. Claims and scope management

Several sentences imply general claims about “science” writ large. To avoid overgeneralization, qualify claims to the sample and fields studied, reserving broader claims as conjectures for future work. Where you invoke system‑level incentives (funding, publication norms), consider adding 1–2 citations from science‑studies/science‑policy to anchor these statements.

________________________________________

7. Small additions on ethics and procedures

Add the incentive amount and form (gift card/transfer) in the Methods to the first paragraph that introduces the interview, to keep all participation details consolidated. Confirm whether participants could review their transcripts (member reflection) and, if not, state this explicitly.</major></general>

**********

what does this mean?). If published, this will include your full peer review and any attached files.

If you choose “no”, your identity will remain anonymous but your review may still be made public.

**Do you want your identity to be public for this peer review?**  For information about this choice, including consent withdrawal, please see our Privacy Policy

Reviewer #1: No

Reviewer #2: **Yes:** Daiki Nakamura

**********

[NOTE: If reviewer comments were submitted as an attachment file, they will be attached to this email and accessible via the submission site. Please log into your account, locate the manuscript record, and check for the action link "View Attachments". If this link does not appear, there are no attachment files.]

While revising your submission, please upload your figure files to the Preflight Analysis and Conversion Engine (PACE) digital diagnostic tool, https://pacev2.apexcovantage.com/. PACE helps ensure that figures meet PLOS requirements. To use PACE, you must first register as a user. Registration is free. Then, login and navigate to the UPLOAD tab, where you will find detailed instructions on how to use the tool. If you encounter any issues or have any questions when using PACE, please email PLOS at figures@plos.org. Please note that Supporting Information files do not need this step.

# Author comment: Author response to Decision Letter 1

**DOI:** 10.1371/journal.pone.0346386.r002

13 Feb 2026

Please find a detailed response to all reviewer comments attached as a PDF, marked: "response to reviewers."

Attachment Submitted filename: Response to Reviewers.pdf

# Peer review: Decision Letter 1

**DOI:** 10.1371/journal.pone.0346386.r003

**Authors:** Katrien Janin

8 May 2026

Dear Dr. James,

Thank you for submitting your manuscript to PLOS ONE. After careful consideration, we feel that it has merit but does not fully meet PLOS ONE’s publication criteria as it currently stands. Therefore, we invite you to submit a revised version of the manuscript that addresses the points raised during the review process.

Your manuscript has now been re‑assessed. While the reviewer acknowledge the improvements, they’ve also noted a few remaining points that need your attention. Please take these points into careful consideration when preparing your revised manuscript and your point‑by‑point response.

Please submit your revised manuscript by Jun 21 2026 11:59PM. If you will need more time than this to complete your revisions, please reply to this message or contact the journal office at plosone@plos.org. When you're ready to submit your revision, log on to https://www.editorialmanager.com/pone/ and select the 'Submissions Needing Revision' folder to locate your manuscript file.

- A letter that responds to each point raised by the academic editor and reviewer(s). You should upload this letter as a separate file labeled 'Response to Reviewers'.
- A marked-up copy of your manuscript that highlights changes made to the original version. You should upload this as a separate file labeled 'Revised Manuscript with Track Changes'.
- An unmarked version of your revised paper without tracked changes. You should upload this as a separate file labeled 'Manuscript'.

If you would like to make changes to your financial disclosure, please include your updated statement in your cover letter. Guidelines for resubmitting your figure files are available below the reviewer comments at the end of this letter.

If applicable, we recommend that you deposit your laboratory protocols in protocols.io to enhance the reproducibility of your results. Protocols.io assigns your protocol its own identifier (DOI) so that it can be cited independently in the future. For instructions see: https://journals.plos.org/plosone/s/submission-guidelines#loc-laboratory-protocols. Additionally, PLOS ONE offers an option for publishing peer-reviewed Lab Protocol articles, which describe protocols hosted on protocols.io. Read more information on sharing protocols at https://plos.org/protocols?utm_medium=editorial-email&utm_source=authorletters&utm_campaign=protocols.

As the corresponding author, your ORCID iD is verified in the submission system and will appear in the published article. PLOS supports the use of ORCID, and we encourage all coauthors to register for an ORCID iD and use it as well. Please encourage your coauthors to verify their ORCID iD within the submission system before final acceptance, as unverified ORCID iDs will not appear in the published article. *Only*  the individual author can complete the verification step; PLOS staff *cannot*  verify ORCID iDs on behalf of authors.

We look forward to receiving your revised manuscript.

Kind regards,

Katrien G. Janin, PhD

Staff Editor

PLOS One

Journal Requirements:

If the reviewer comments include a recommendation to cite specific previously published works, please review and evaluate these publications to determine whether they are relevant and should be cited. There is no requirement to cite these works unless the editor has indicated otherwise.

Please review your reference list to ensure that it is complete and correct. If you have cited papers that have been retracted, please include the rationale for doing so in the manuscript text, or remove these references and replace them with relevant current references. Any changes to the reference list should be mentioned in the rebuttal letter that accompanies your revised manuscript. If you need to cite a retracted article, indicate the article’s retracted status in the References list and also include a citation and full reference for the retraction notice.

[Note: HTML markup is below. Please do not edit.]

Reviewers' comments:

Reviewer’s Responses to Questions

**Comments to the Author**

Reviewer #3: (No Response)

**********

2. Is the manuscript technically sound, and do the data support the conclusions?

Reviewer #3: Partly

**********

3. Has the statistical analysis been performed appropriately and rigorously?

Reviewer #3: I Don’t Know

**********

4. Have the authors made all data underlying the findings in their manuscript fully available?

The PLOS Data policy

Reviewer #3: Yes

**********

5. Is the manuscript presented in an intelligible fashion and written in standard English?

Reviewer #3: Yes

**********

Reviewer #3: I appreciate you pursuing such an interesting line of inquiry. It was a pleasure to review your work, and I found the content to be very engaging.

This paper has already passed its first review. It’s been improved a lot since the first version, and the methodology is now much more rigorous and solid.

The paper has improved and become more sophisticated, however, I noticed a few points in the latest version that might a bit tricky for readers. Here are some suggestions to make it even better.

[Although the authors explicitly state in their theoretical foundations that they adopt a Critical Realism paradigm, there is a notable absence of an in-depth discussion utilizing its core ontology—the three stratified domains (the Empirical, the Actual, and the Real)—when interpreting the findings. The significance of the five themes derived through Reflexive Thematic Analysis (RTA) in this study becomes much more multidimensional and profound when interpreted through this three-tiered structure of Critical Realism. For instance, the anxiety and identity threats experienced by participants (Theme 4) can be mapped to the observable 'domain of the empirical'; the exploratory research and HARKing practices occurring in the field (Themes 2, 3, and 5) to the 'domain of the actual'; and the cultural master narrative of science (CMNOS, Theme 1), which acts as the underlying structural force continuously generating these experiences and practices, to the 'domain of the real'. Therefore, building upon the stated paradigm, it would significantly strengthen the paper if the authors could expand the Discussion section by deeply reinterpreting the inductively derived RTA themes through the lens of Critical Realism’s three-tiered structure. Furthermore, presenting a visualized conceptual model that clearly illustrates these multi-layered interactions would significantly strengthen the study’s methodological congruence and theoretical completeness.]

I am not suggesting that Critical Realism (CR) should be used as a codebook for Reflexive Thematic Analysis (RTA). Rather, my point is that the data and themes derived through RTA need to be explained and interpreted within the theoretical framework of CR. Since the authors explicitly stated that they adopted a Critical Realist perspective, it seems necessary to address this connection.

1. The author states on page 4 that “it is important to recognize that, regardless of the NoS formulation employed, implicitly-developed views about science often conflict with established tenets of NoS”.

From the perspective of the Family Resemblance Approach (FRA), the participants’ responses could interpreted not as ‘conflicts’ with a master narrative, but as legitimate disciplinary variations reflecting ‘family resemblances’ across different scientific fields or theme. By framing these nuances simply as ‘conflicts’ regardless of the formulation used, the study risks oversimplifying the phenomena.

The theoretical coherence of the manuscript could be further enhanced by refining the framing as follows:

① Minimize or delete unnecessary references to alternative perspectives like FRA or FoS if they are not actively used as the primary analytical lens.

② Remove the phrase “regardless of the NoS formulation employed”, as it creates a contradiction when the analysis subsequently relies on specific tenets.

③ Instead, explicitly state that this study adopts the Consensus View (CV) as its primary analytical framework. (I have observed that the authors are relying exclusively on the tenets of CV (Consensus View) in their interpretation of the findings. It was for this reason that I put forward the aforementioned suggestions.)

2. Additionally, in the same sentence, it is logically unclear why implicitly-acquired views must be compared specifically against the CV, “regardless of the Nos formulation employed.” If one adopts an alternative formulation such as the FRA, a deviation from the CV’s tenets is not a ‘conflict’ to be corrected, but a legitimate epistemological variation. By insisting on a comparison with ‘established tenets (which function here as the CV)”, the author inadvertently privileges the CV over other formulations.

3. The author presents a pivotal theoretical argument in lines 690-692: “This is well aligned with the NoS, which describes science as a creative and social process. However, this is not conserved in the CMNoS, which associates “good” science with being strictly structured and objective.” While this conclusion is central to the study, I find a significant structural issue regarding its substantiation.

The empirical foundation for this claim—specifically regarding the prioritization of intuition over formal hypotheses—is located several pages earlier in the Results section (near lines 525–526). By failing to provide a summarized re-citation or an explicit cross-reference within the Discussion section, the author forces the reader to rely solely on memory or engage in the inconvenient task of constantly flipping back and forth between pages to verify the argument.

The structural that strictly segregates the ‘results’, discussion’, and ‘implications’ poses significant challenges for the reader. If this structure is maintained, the discussion would greatly benefit from explicit cross-referencing to the specific portions of the results that support each statement during the narrative of the discussion and implications. A lack of immediate connectivity between the participants’ narratives and the theoretical analysis may cause the author’s logic to be perceived as an ‘interpretive leap’, which could potentially obscure the methodological rigor that the authors have achieved.

4. The author provides an extensive review of the Consensus View (CV) of the nature of science (NOS) in chapter 2, establishing it as a primary analytical framework. However, there is a significant lack of clarity regarding how this framework is practically applied to the data and how it interacts with the newly proposed CMNoS.

① The CV consists of 7 to 8 distinct tenets (e.g., subjectivity, creativity, social-cultural embeddedness). The CMNoS, as described in this study, appears to focus almost exclusively on the ‘myth of a single scientific method’ (specifically, the hypothetico-deductive model). This raises a critical question: Is CMNoS merely a ‘misconception’ of a single CV tenet regarding the diversity of scientific methods, or it a truly a ‘master narrative’ that encompasses the entire NOS? If it is the former, it would be helpful to elaborate on why it is treated as a grand cultural narrative rather than a localized misconception within the NOS framework.

② In the discussion, the author introduces other tenets such as ‘imagination and creativity’ to critique the rigidity of the CMNoS. This suggests that the analysis is not limited to ‘methodological diversity’ but involves multiple CV tenets. The author needs to explicitly specify which of the 7-8 tenets are being utilized as analytical lenses and which are excluded (and why).

③ I have a fundamental concern regarding whether the CMNoS, as currently presented, a possesses the functional properties of a rigorous analytical framework or if it is merely a single-sentence definition used as a general guideline during the reflexive thematic analysis. If the CMNoS serves only as a broad interpretive lens rather than a structured system for data categorization, the current usage of the term 'framework' might lead to reader expectations regarding methodological structure that are not fully realized in the text. Providing a conceptual map could resolve this. To justify the use of this term, the author should make a concerted effort to visualize the CMNoS, providing a structural map or a model that clarifies its internal dimensions and how it systematically interacts with the raw data, rather than leaving it as an ambiguous conceptual label.

5. I believe the authors' choice to adopt Reflexive Thematic Analysis (RTA) to answer their research questions is a highly appropriate methodological decision. My critique by no means implies forcing the Nature of Science (NOS) to be used as a rigid "deductive codebook" at the expense of undermining RTA’s flexible epistemological principles. While RTA is undoubtedly an excellent methodology for reading the broader context of the data and emergently weaving together meanings, the analytical step of identifying or mapping that "this participant’s utterance demonstrates a specific NOS perspective" typically necessitates a clear analytical baseline to ground such interpretive judgments transparently.

In other words, to determine that a specific NOS tenet has manifested in the raw data—which serves as the foundational material for deriving overarching themes in RTA—there must be an antecedent operational definition of the criteria the researchers used to examine the data. Therefore, to prevent arbitrariness in qualitative interpretation and to ensure analytical transparency and rigor, I strongly recommend that the authors explicitly detail in the Data Analysis section the specific standards they utilized when identifying and mapping particular Consensus View (CV) tenets within the data.

**********

what does this mean?). If published, this will include your full peer review and any attached files.

If you choose “no”, your identity will remain anonymous but your review may still be made public.

**Do you want your identity to be public for this peer review?**  For information about this choice, including consent withdrawal, please see our Privacy Policy

Reviewer #3: No

**********

[NOTE: If reviewer comments were submitted as an attachment file, they will be attached to this email and accessible via the submission site. Please log into your account, locate the manuscript record, and check for the action link "View Attachments". If this link does not appear, there are no attachment files.]

To ensure your figures meet our technical requirements, please review our figure guidelines: https://journals.plos.org/plosone/s/figures

You may also use PLOS’s free figure tool, NAAS, to help you prepare publication quality figures: https://journals.plos.org/plosone/s/figures#loc-tools-for-figure-preparation.

NAAS will assess whether your figures meet our technical requirements by comparing each figure against our figure specifications.

Attachment Submitted filename: review.docx

# Author comment: Author response to Decision Letter 2

**DOI:** 10.1371/journal.pone.0346386.r004

1 Jul 2026

Reviewer response included with resubmission as PDF upload entitled "Response to Reviewers"

Attachment Submitted filename: Response_to_Reviewers_auresp_2.pdf

# Peer review: Decision Letter 2

**DOI:** 10.1371/journal.pone.0346386.r005

**Authors:** Shengqian Sun

28 Jul 2026

“The hypothesis becomes forced”:  Cultural narratives about deductive hypothesis testing influence scientists’ self-perception and actions

PONE-D-25-36217R2

Dear Dr. James,

We’re pleased to inform you that your manuscript has been judged scientifically suitable for publication and will be formally accepted for publication once it meets all outstanding technical requirements.

Within one week, you’ll receive an e-mail detailing the required amendments. When these have been addressed, you’ll receive a formal acceptance letter and your manuscript will be scheduled for publication.

An invoice will be generated when your article is formally accepted. Please note, if your institution has a publishing partnership with PLOS and your article meets the relevant criteria, all or part of your publication costs will be covered. Please make sure your user information is up-to-date by logging into Editorial Manager at Editorial Manager® and clicking the ‘Update My Information' link at the top of the page. For questions related to billing, please contact billing support.

If your institution or institutions have a press office, please notify them about your upcoming paper to help maximize its impact. If they’ll be preparing press materials, please inform our press team as soon as possible -- no later than 48 hours after receiving the formal acceptance. Your manuscript will remain under strict press embargo until 2 pm Eastern Time on the date of publication. For more information, please contact onepress@plos.org.

Kind regards,

Shengqian Sun

Academic Editor

PLOS One

Additional Editor Comments (optional):

Reviewers' comments:

Reviewer’s Responses to Questions

**Comments to the Author**

Reviewer #3: All comments have been addressed

**********

2. Is the manuscript technically sound, and do the data support the conclusions?

Reviewer #3: Yes

**********

3. Has the statistical analysis been performed appropriately and rigorously?

Reviewer #3: Yes

**********

4. Have the authors made all data underlying the findings in their manuscript fully available?

The PLOS Data policy

Reviewer #3: Yes

**********

5. Is the manuscript presented in an intelligible fashion and written in standard English?

Reviewer #3: Yes

**********

Reviewer #3: (No Response)

**********

what does this mean?). If published, this will include your full peer review and any attached files.

If you choose “no”, your identity will remain anonymous but your review may still be made public.

**Do you want your identity to be public for this peer review?**  For information about this choice, including consent withdrawal, please see our Privacy Policy

Reviewer #3: No

**********

Attachment Submitted filename: Reviewer Comments.docx

# Editor report: Acceptance letter

**DOI:** 10.1371/journal.pone.0346386.r006

**Authors:** Shengqian Sun

PONE-D-25-36217R2

PLOS One

Dear Dr. James,

I'm pleased to inform you that your manuscript has been deemed suitable for publication in PLOS One. Congratulations! Your manuscript is now being handed over to our production team.

At this stage, our production department will prepare your paper for publication. This includes ensuring the following:

* All references, tables, and figures are properly cited

* All relevant supporting information is included in the manuscript submission,

* There are no issues that prevent the paper from being properly typeset

You will receive further instructions from the production team, including instructions on how to review your proof when it is ready. Please keep in mind that we are working through a large volume of accepted articles, so please give us a few days to review your paper and let you know the next and final steps.

Lastly, if your institution or institutions have a press office, please let them know about your upcoming paper now to help maximize its impact. If they'll be preparing press materials, please inform our press team within the next 48 hours. Your manuscript will remain under strict press embargo until 2 pm Eastern Time on the date of publication. For more information, please contact onepress@plos.org.

You will receive an invoice from PLOS for your publication fee after your manuscript has reached the completed accept phase. If you receive an email requesting payment before acceptance or for any other service, this may be a phishing scheme. Learn how to identify phishing emails and protect your accounts at https://explore.plos.org/phishing.

If we can help with anything else, please email us at customercare@plos.org.

Thank you for submitting your work to PLOS One and supporting open access.

Kind regards,

PLOS ONE Editorial Office Staff

on behalf of

Dr. Shengqian Sun

Academic Editor

PLOS One
